# Supplementary material for: Synthesis of a New β-Galactosidase Inhibitor Displaying Pharmacological Chaperone Properties for GM1 Gangliosidosis
Source: Molecules. 2022 Jun 22;27(13):4008. doi: 10.3390/molecules27134008 (PMC9268699; doi:10.3390/molecules27134008)
Supplement: Supplementary file 1 [file molecules-27-04008-s001.zip › molecules-1776877-supplementary.pdf]

Synthesis of a new β-galactosidase inhibitor displaying pharmacological chaperone properties for GM1 gangliosidosis

Francesca Clemente [1,*] Macarena Martínez-Bailén [1,2], Camilla Matassini [1], Amelia Morrone [3,4], Silvia Falliano [4], Anna Caciotti [4], Paolo Paoli [5], Andrea Goti [1] and Francesca Cardona [1,*]

[1] Dipartimento di Chimica "Ugo Schiff" (DICUS), Università di Firenze, Via Della Lastruccia 3-13, 50019 Sesto Fiorentino, Italy; macarena.martinez@iiq.csic.es (M.M.-B.); camilla.matassini@unifi.it (C.M.); andrea.goti@unifi.it (A.G.); francesca.cardona@unifi.it (F.C.)

[2] Glycosystems Laboratory, Instituto de Investigaciones Químicas (IIQ), CSIC—Universidad de Sevilla, Av. Américo Vespucio 49, 41092 Sevilla, Spain

[3] Department of Neurosciences, Pharmacology and Child Health (NEUROFARBA), University of Florence, Viale Pieraccini n. 24, 50139 Firenze, Italy; amelia.morrone@unifi.it (A. M.)

[4] Laboratory of Molecular Biology of Neurometabolic Diseases, Neuroscience Department, Meyer Children's Hospital, Viale Pieraccini n. 24, 50139 Firenze, Italy; amelia.morrone@unifi.it (A. M.); silvia.falliano@meyer.it (S.F.); anna.caciotti@meyer.it (A.C.)

[5] Dipartimento di Scienze Biomediche Sperimentali e Cliniche "Mario Serio" (DSBSC), University of Florence, Viale Morgagni 50, 50134 Florence, Italy; paolo.paoli@unifi.it (P.P.)

* Correspondence: francesca.clemente@unifi.it (F.C.); francesca.cardona@unifi.it (F.C.)
Tel.: +39-055-4573453 (F.C.); Tel.: +39-055-4573504 (F.C.)

SUPPORTING INFORMATION

Table of contents

^{1}H-NMR spectrum of compound **16**	S01
^{1}H-NMR spectrum of compound **17**	S02
^{1}H-NMR and ^{13}C-NMR spectra of compound **22**	S03-S04
^{1}H-NMR and ^{13}C-NMR spectra of compound **23**	S05-S06
^{1}H-NMR and ^{13}C-NMR spectra of compound **14**	S07-S08
^{1}H-NMR, ^{13}C-NMR and 1D NOESY spectra of compound **15**	S09-S11
^{1}H-NMR and ^{13}C-NMR spectra of compound **10**	S12-S13
^{1}H-NMR and ^{13}C-NMR spectra of compound **12**	S14-S15
^{1}H-NMR and ^{13}C-NMR spectra of compound **24**	S16-S17
^{1}H-NMR and ^{13}C-NMR spectra of compound **25**	S18-S19
^{1}H-NMR and ^{13}C-NMR spectra of compound **26**	S20-S21
^{1}H-NMR and ^{13}C-NMR spectra of compound **27**	S22-S23
^{1}H-NMR and ^{13}C-NMR spectra of compound **28**	S24-S25
^{1}H-NMR and ^{13}C-NMR spectra of compound **29**	S26-S27
^{1}H-NMR, ^{13}C-NMR and 1D NOESY spectra of compound **11**	S28-S30
^{1}H-NMR, ^{13}C-NMR and 1D NOESY spectra of compound **13**	S31-S33
^{1}H-NMR and ^{13}C-NMR spectra of compound **30**	S34-S35
^{1}H-NMR and ^{13}C-NMR spectra of compound **21**	S36-S37
Biological Screening towards Human Lysosomal β-Galactosidase and β-Glucosidase	S38-S39
Kinetic Analysis for compound **12** *vs* β-Gal	S40
Pharmacological Chaperoning Activity	S42-S43

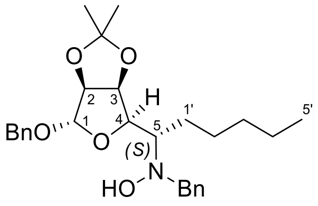

16

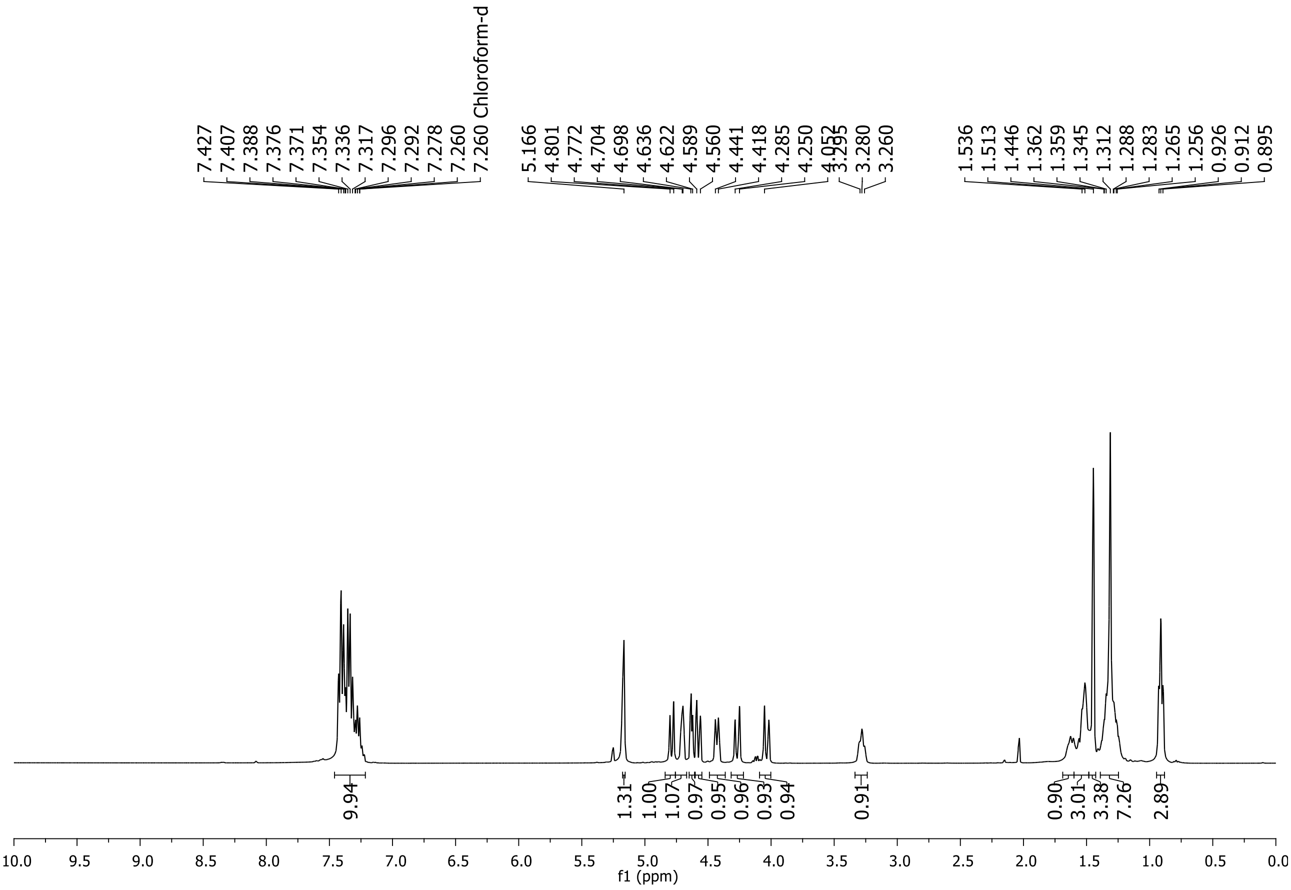

Figure S01. ^{1}H-NMR spectrum of compound **16** (400 MHz, $CDCl_3$).

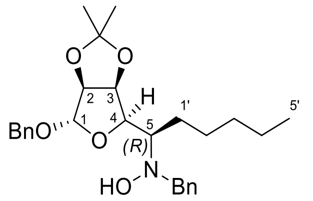

17

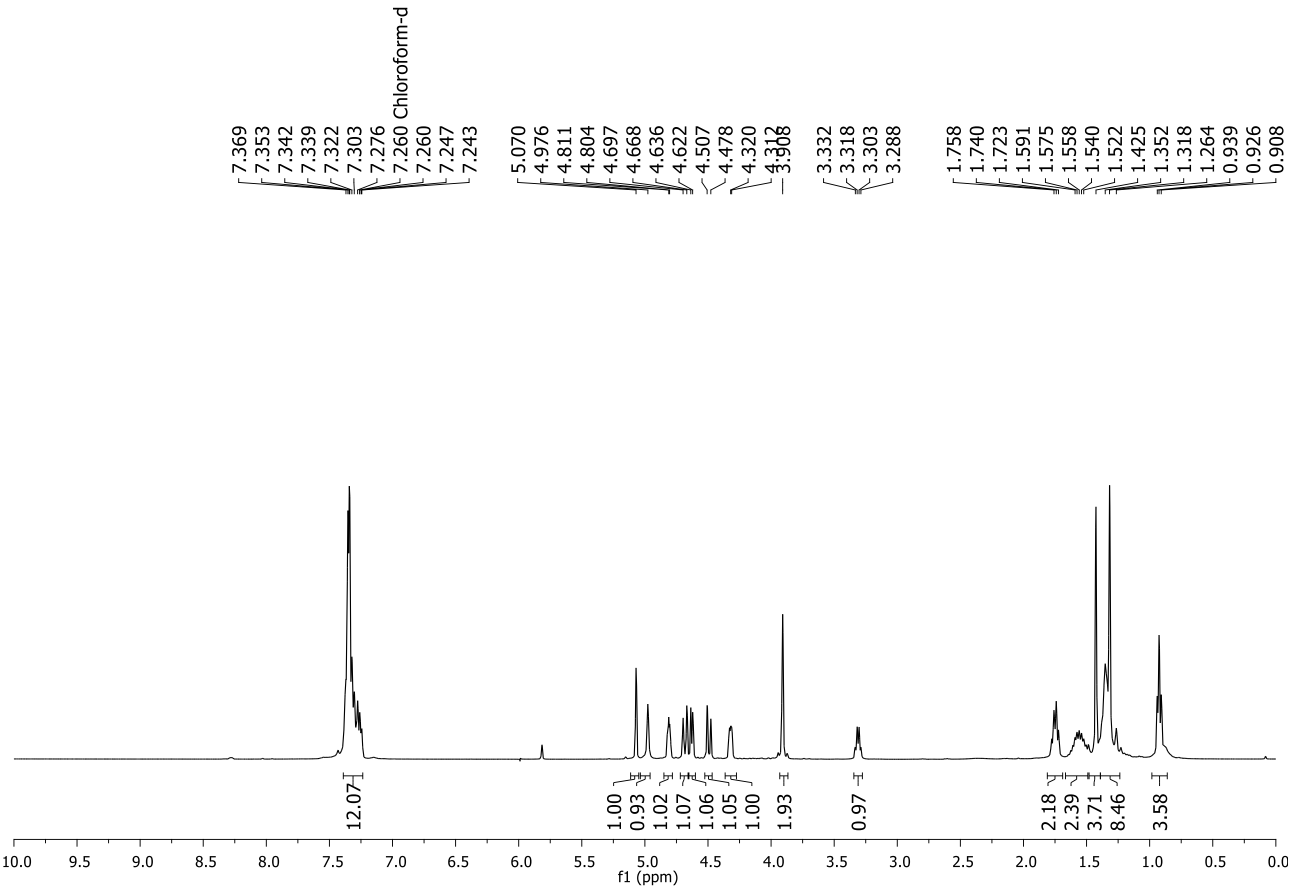

Figure S02. ^{1}H-NMR spectrum of compound **17** (400 MHz, $CDCl_3$).

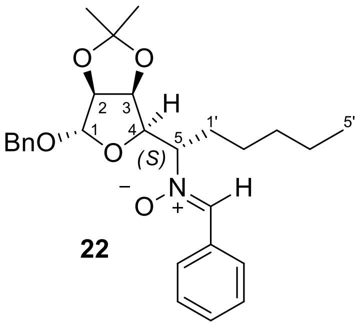

22

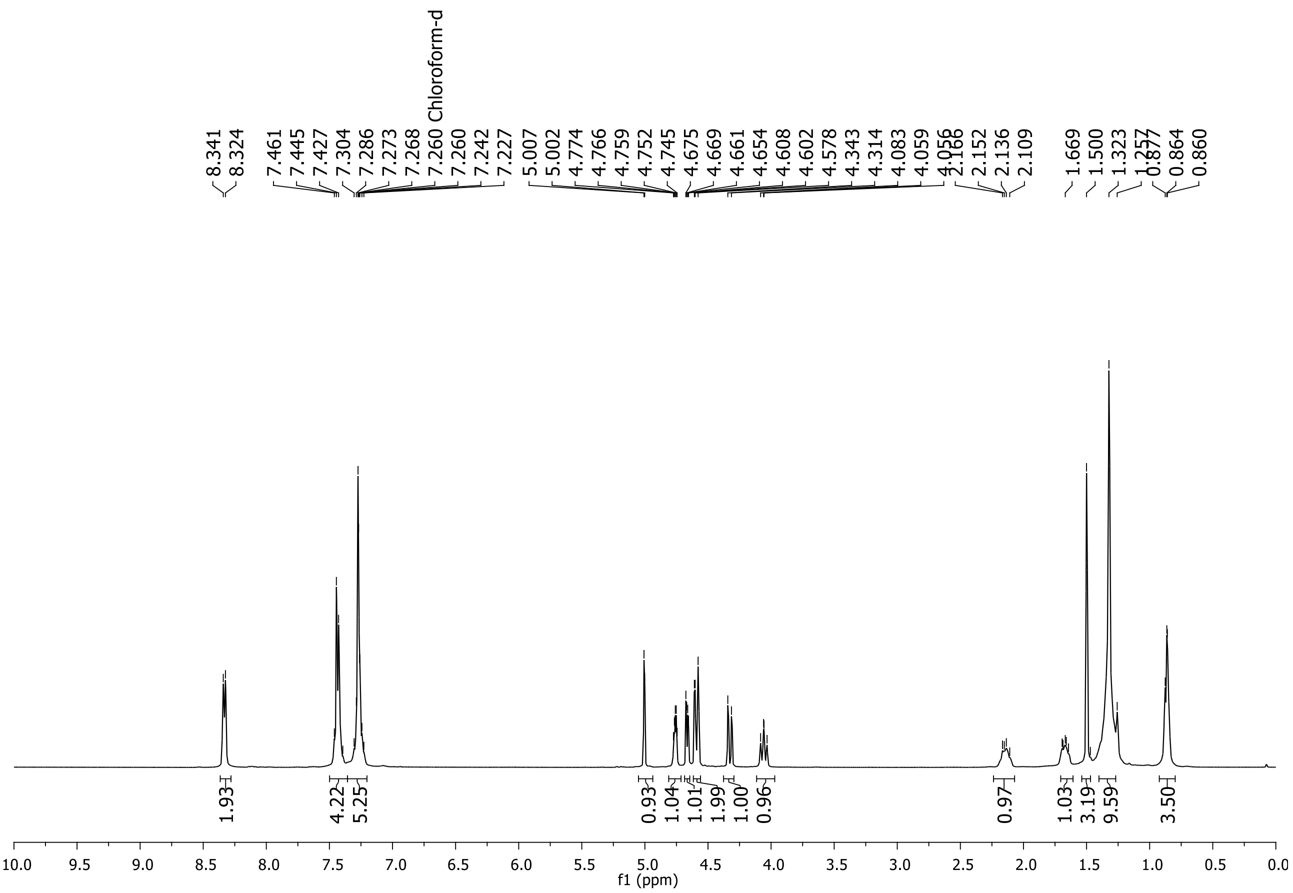

Figure S03. ^{1}H-NMR spectrum of compound **22** (400 MHz, $CDCl_3$).

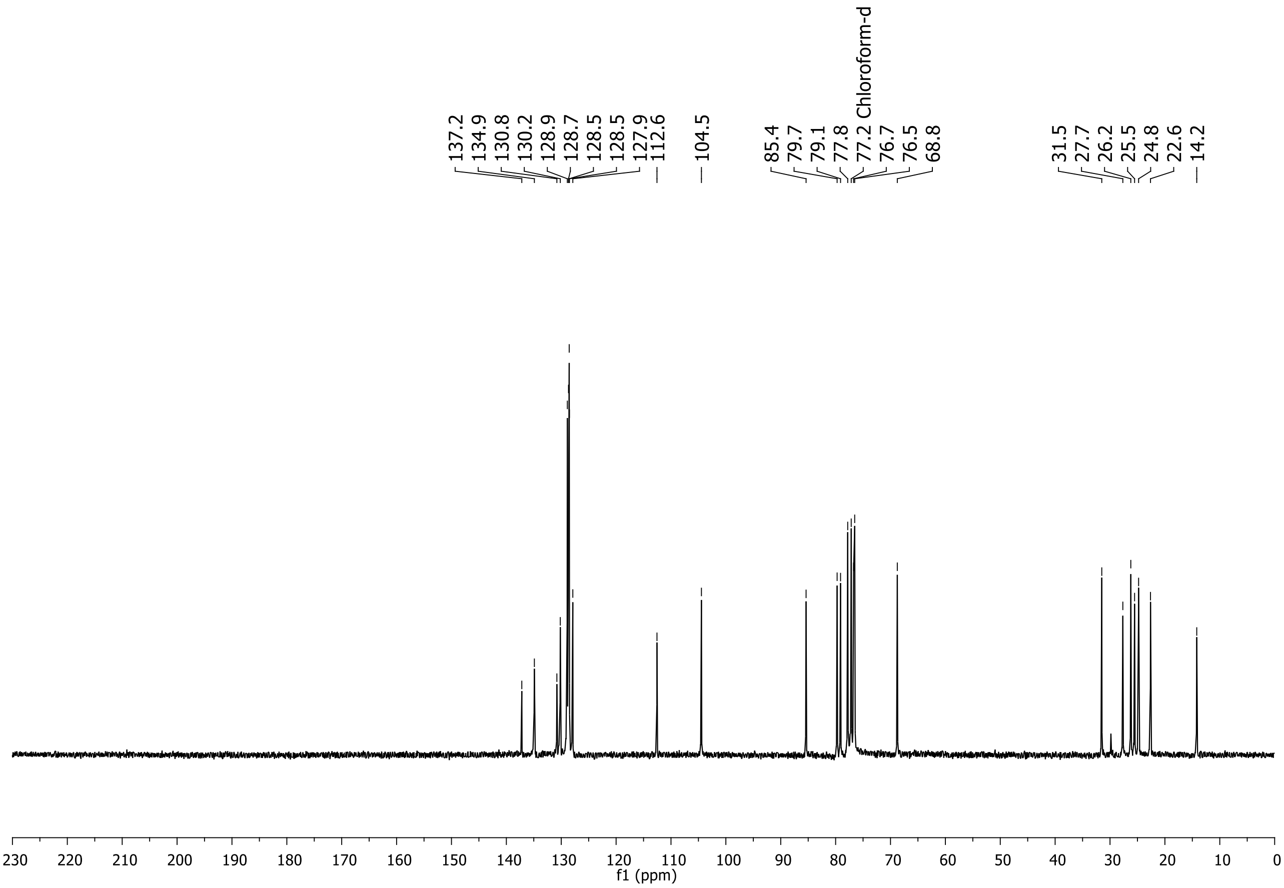

Figure S04. ^{13}C-NMR spectrum of compound **22** (50 MHz, $CDCl_3$).

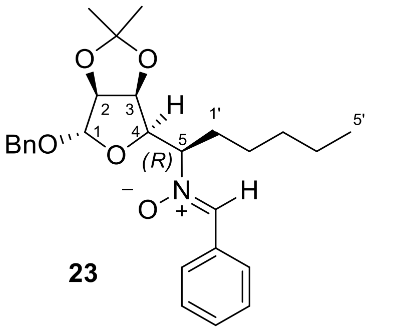

23

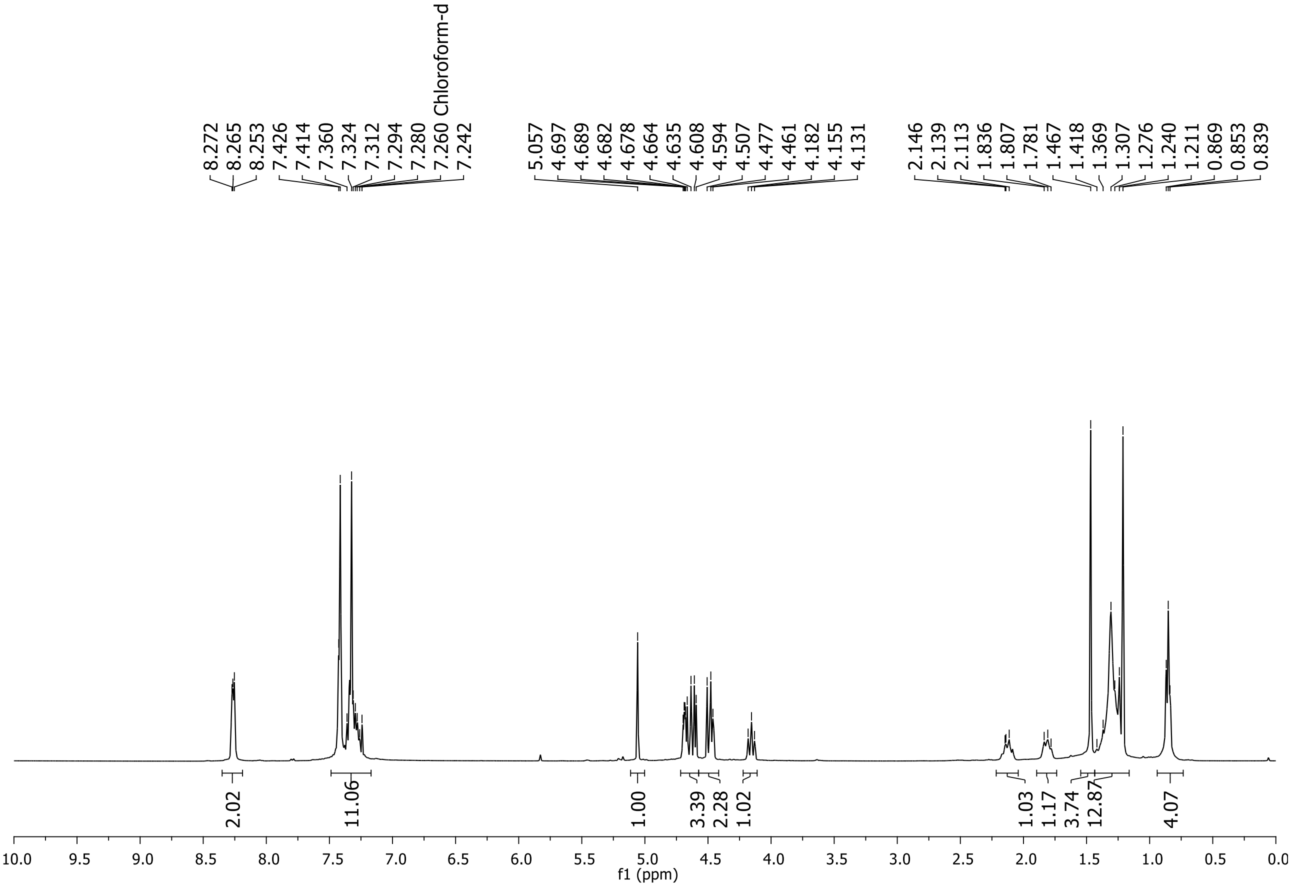

Figure S05. ^{1}H-NMR spectrum of compound **23** (400 MHz, $CDCl_3$).

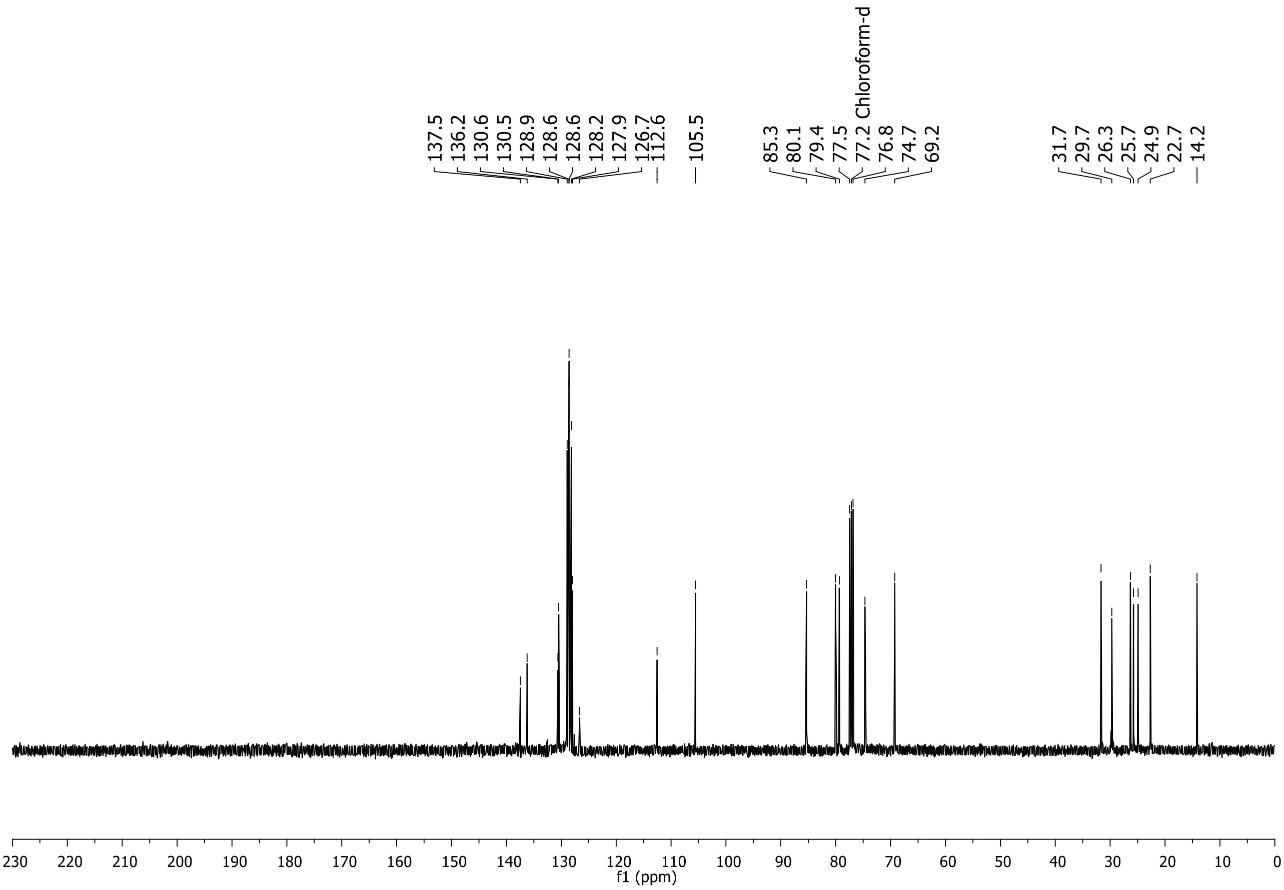

Figure S06. ^{13}C-NMR spectrum of compound **23** (100 MHz, $CDCl_3$).

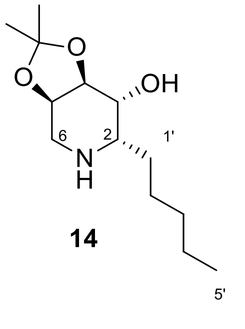

14

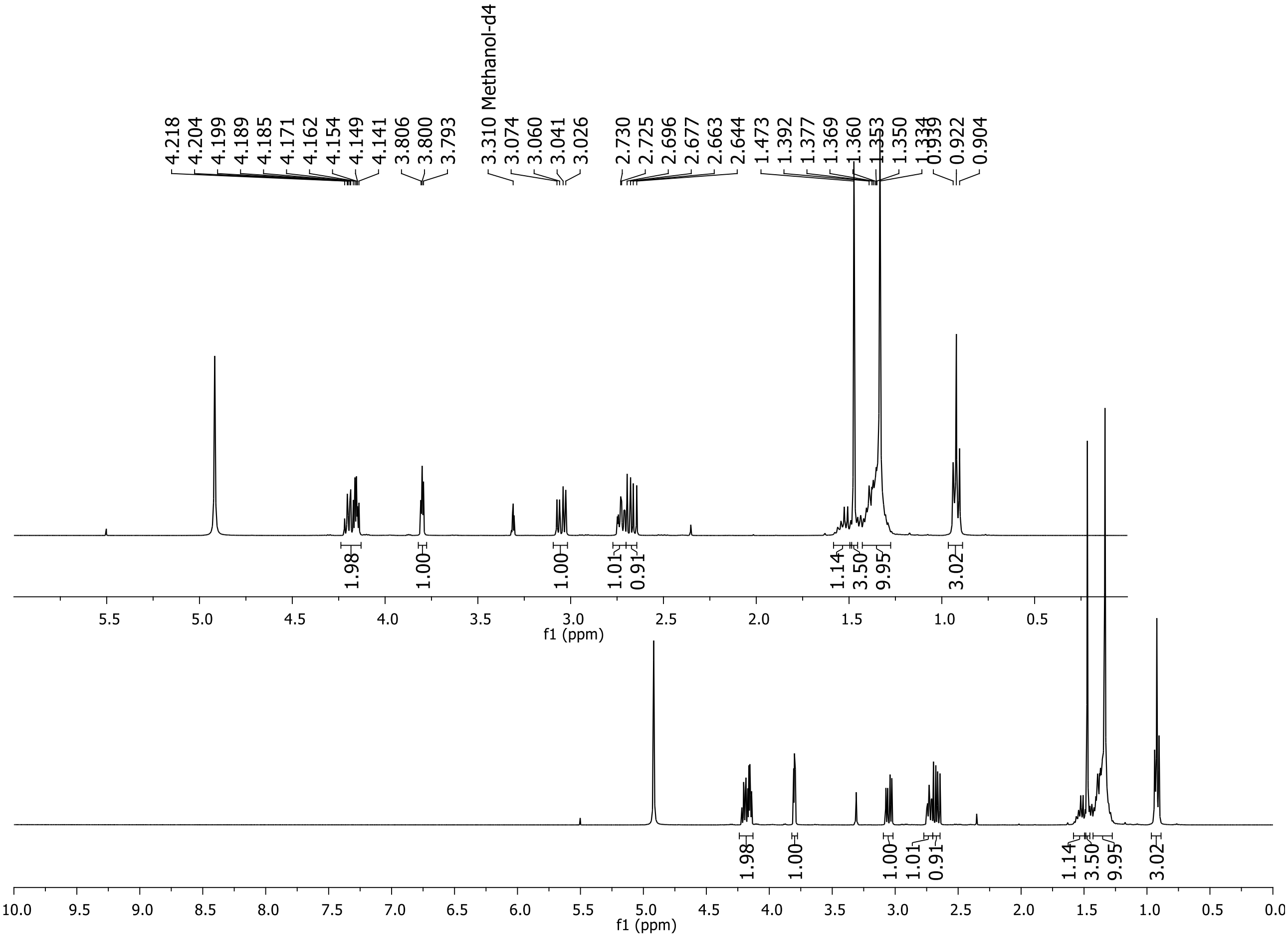

Figure S07. ^{1}H-NMR spectrum of compound **14** (400 MHz, CD_3OD).

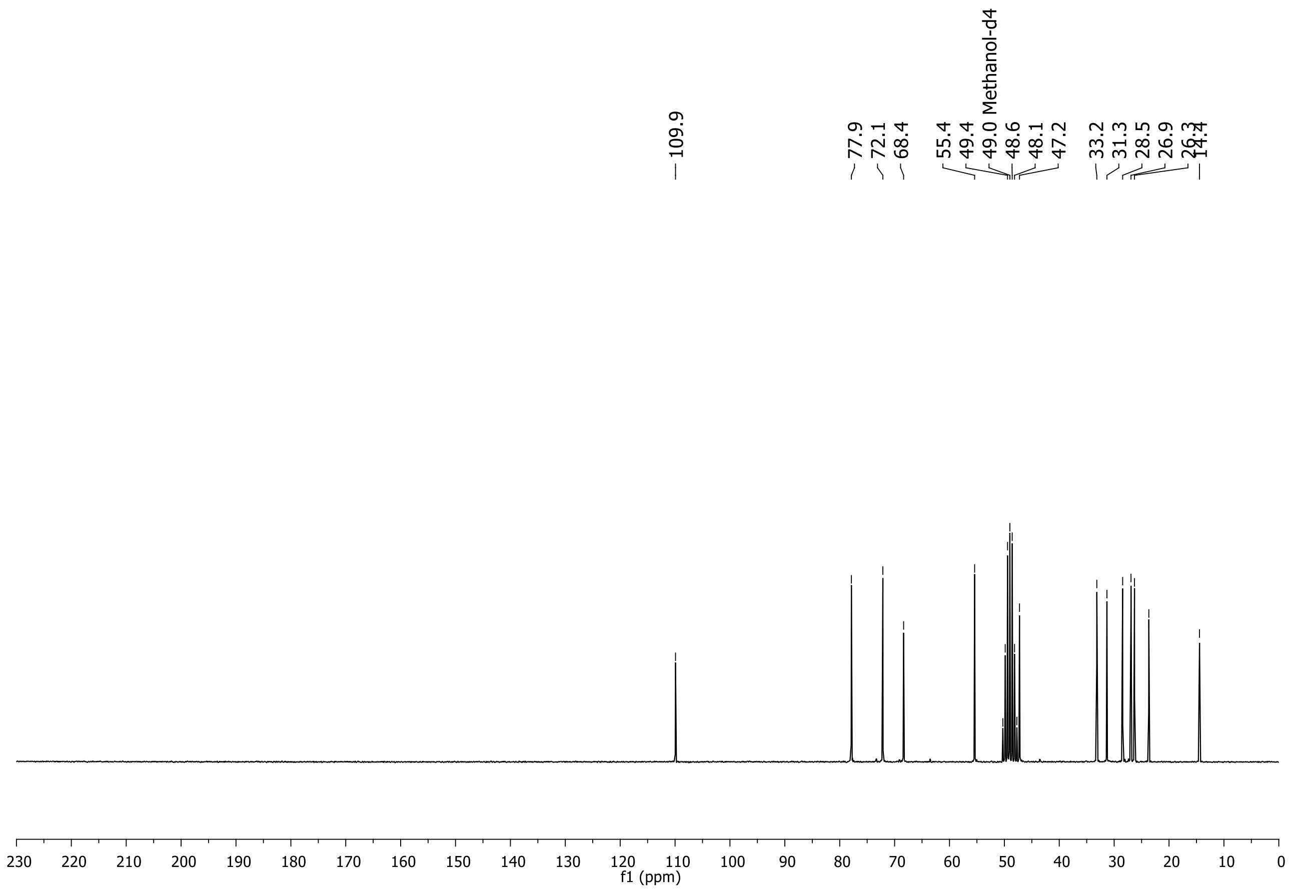

Figure S08. ^{13}C-NMR spectrum of compound **14** (50 MHz, CD_3OD).

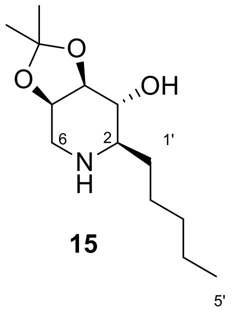

15

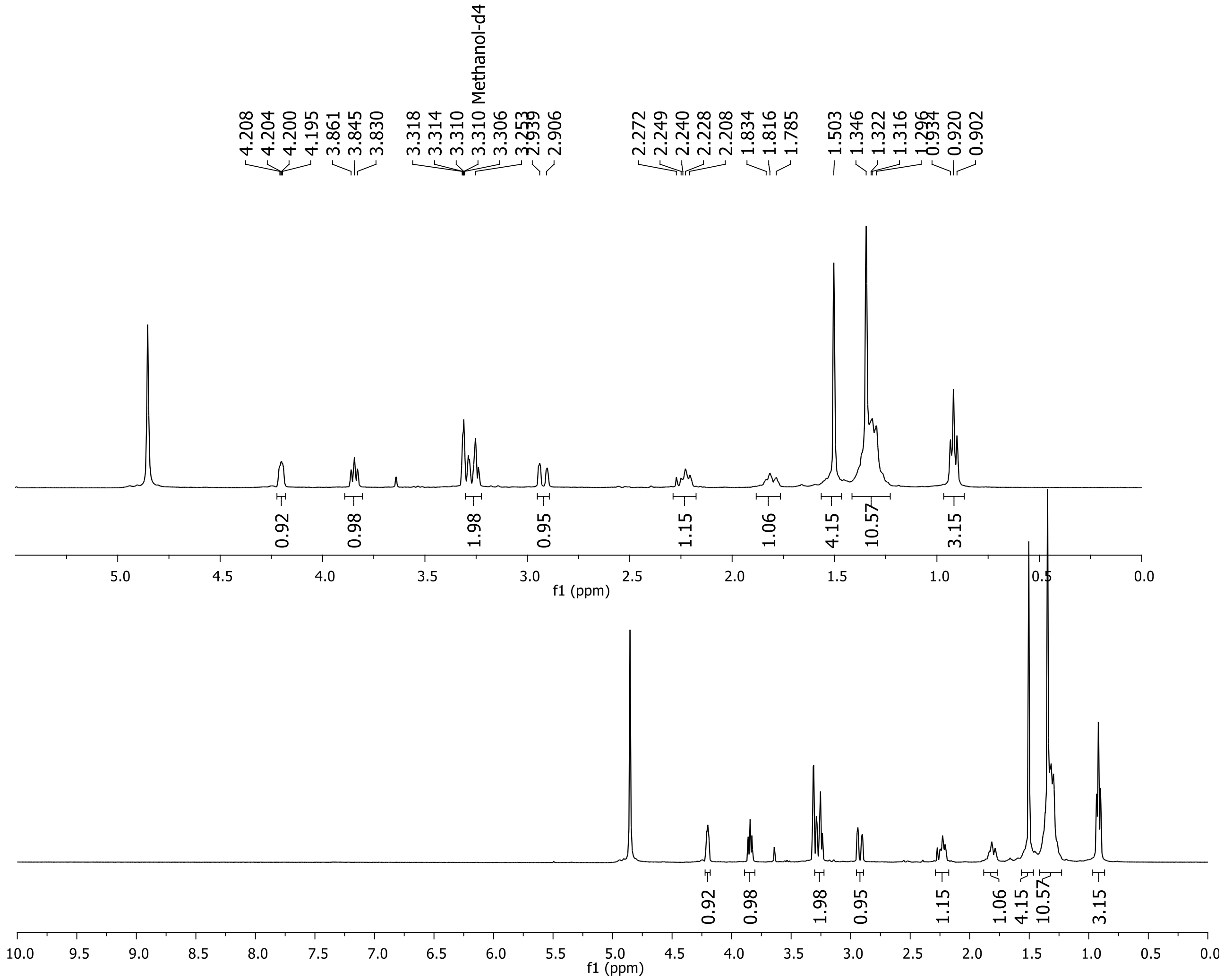

Figure S09. ^{1}H-NMR spectrum of compound **15** (400 MHz, CD_3OD).

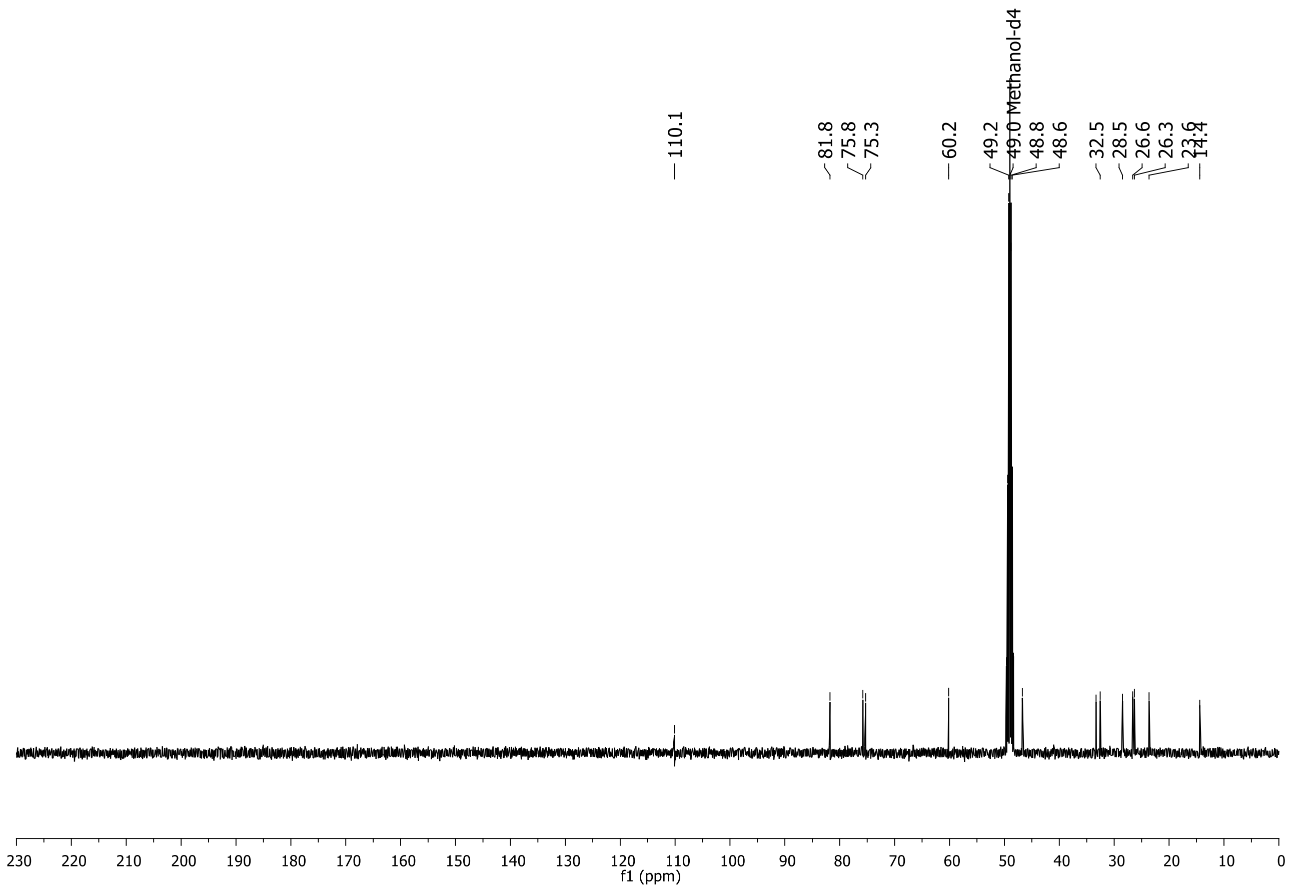

Figure S10. ^{13}C-NMR spectrum of compound **15** (100 MHz, CD_3OD).

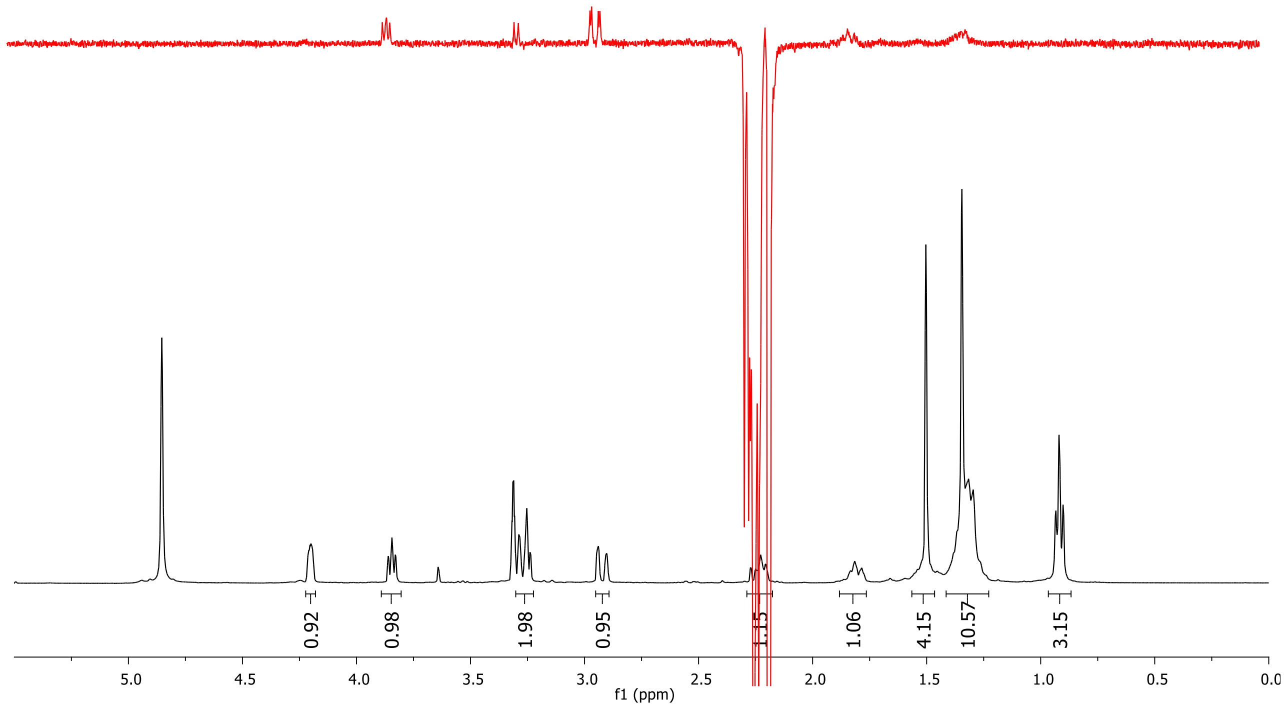

Figure S11: [1]D-NOESY spectra performed on compound **15**. Irradiation of H-2 gave a NOE at H-4 and H_b-6.

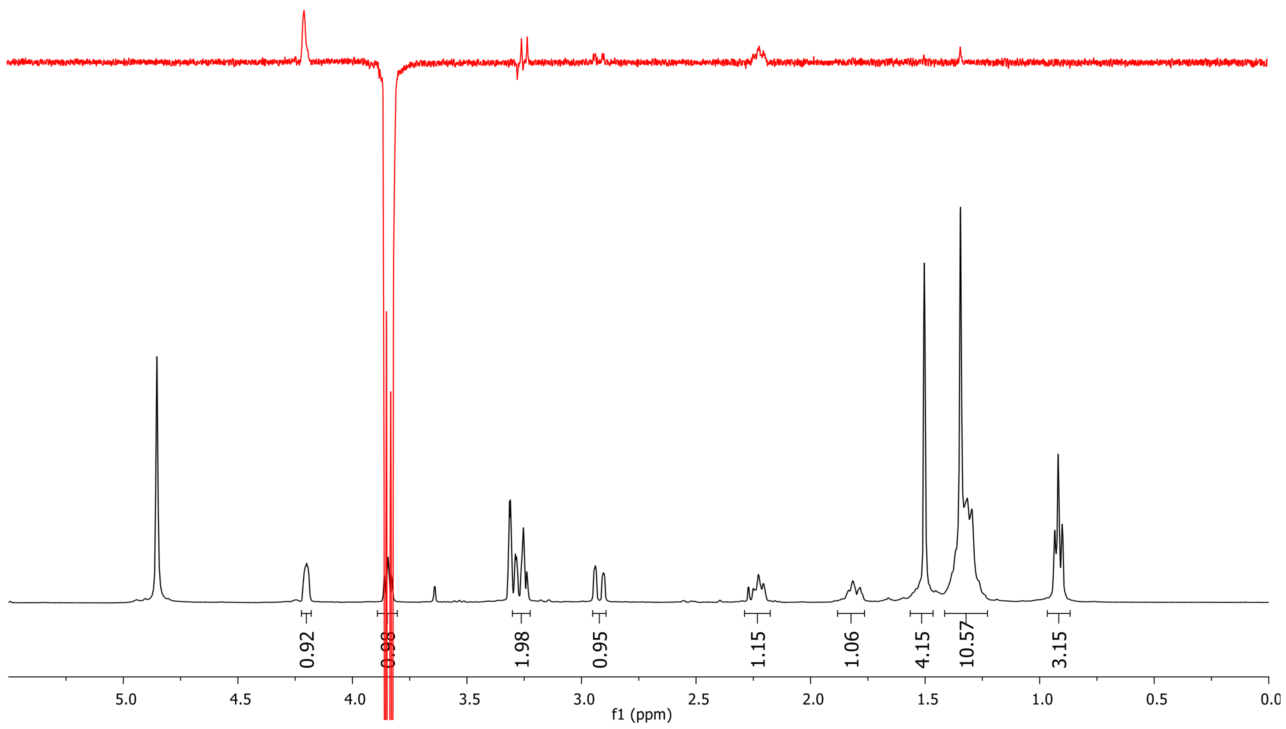

Figure S12: [1]D-NOESY spectra performed on compounds **15.** Irradiation of H-4 gave a NOE at H-2 and H_b-6.

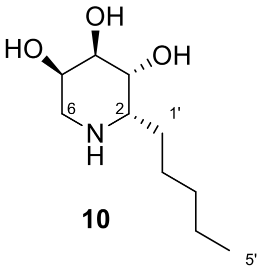

10

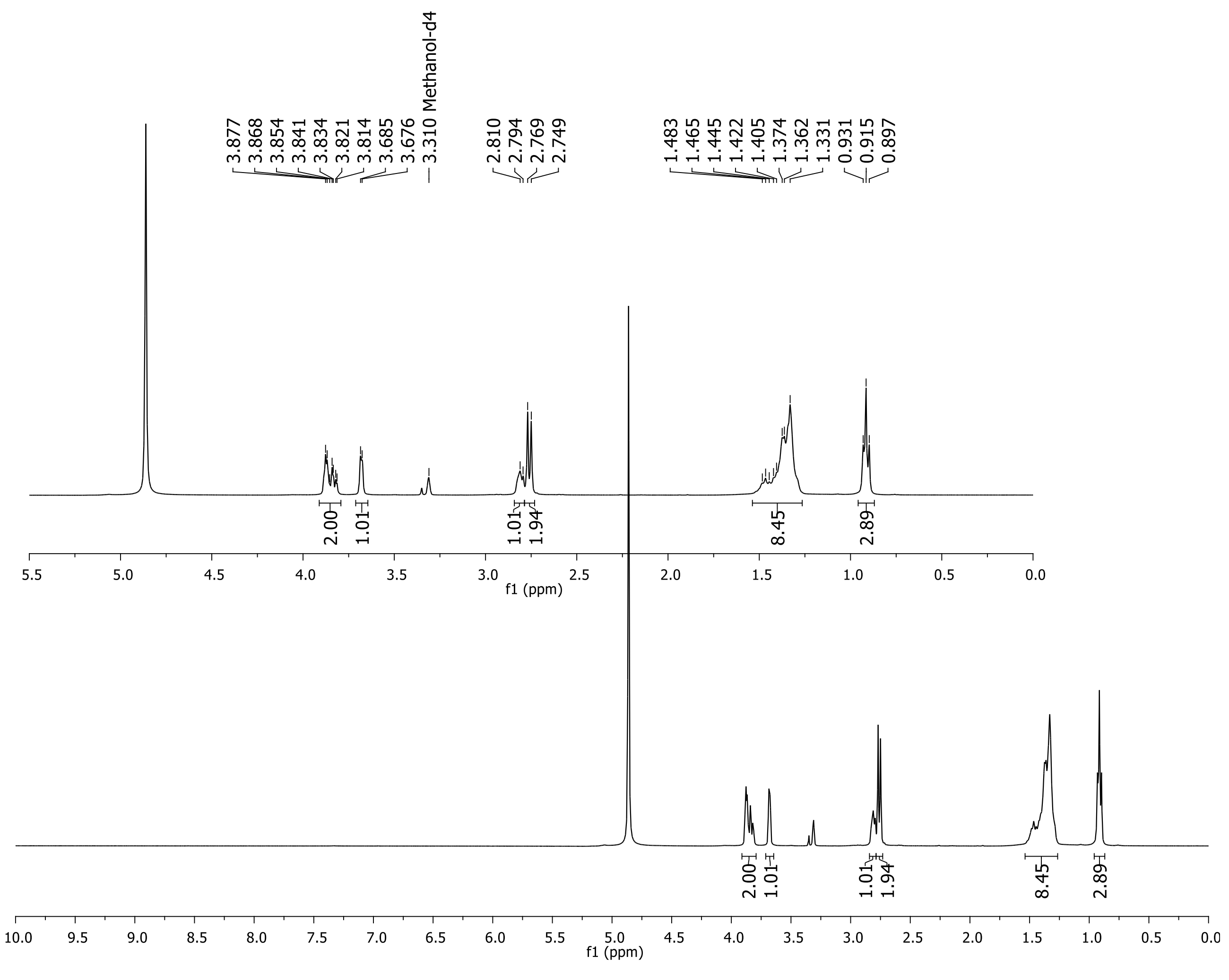

Figure S13. ^{1}H-NMR spectrum of compound **10** (400 MHz, CD_3OD).

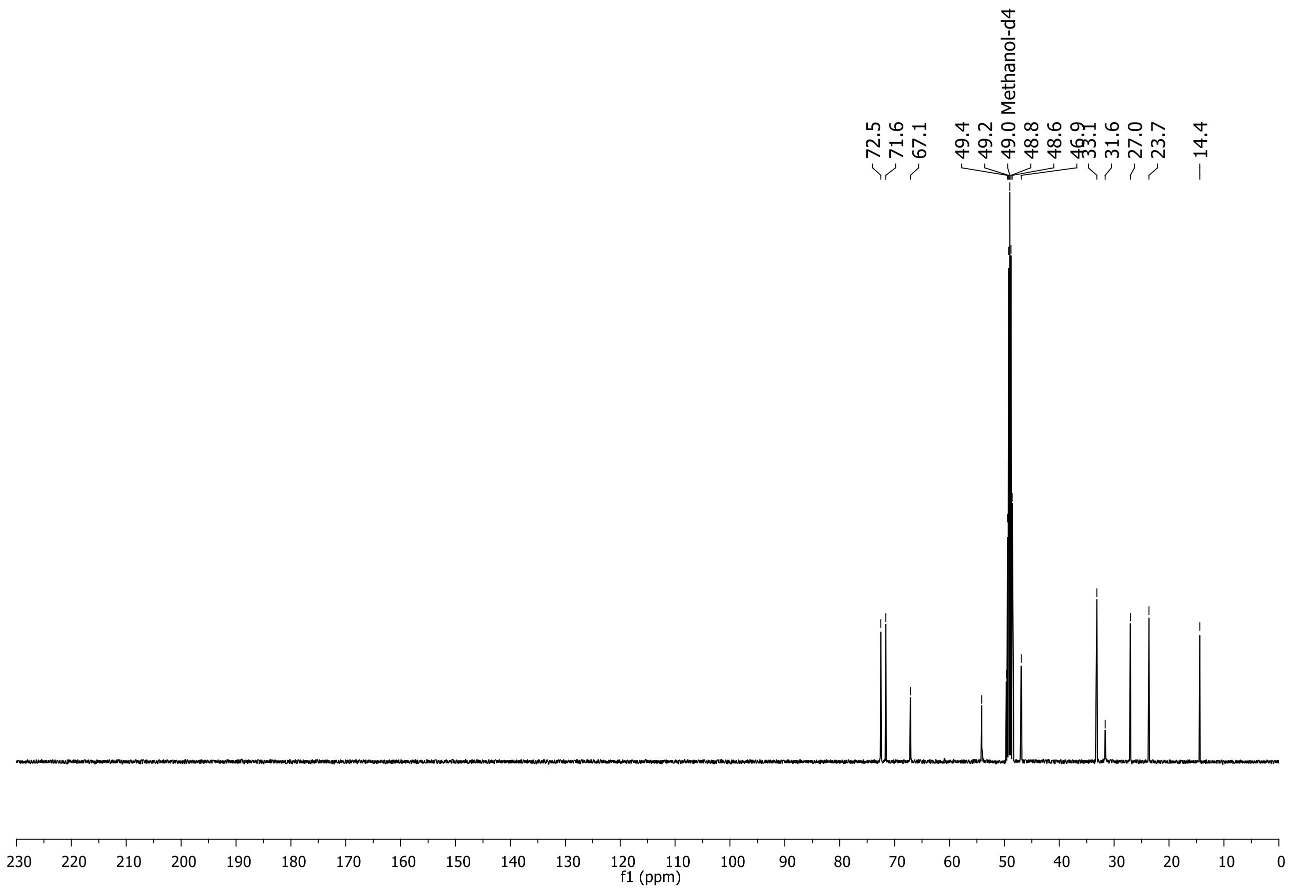

Figure S14. ^{13}C-NMR spectrum of compound **10** (100 MHz, CD_3OD).

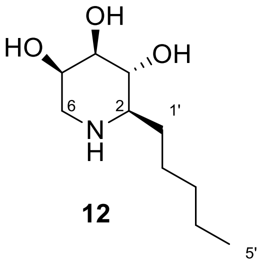

12

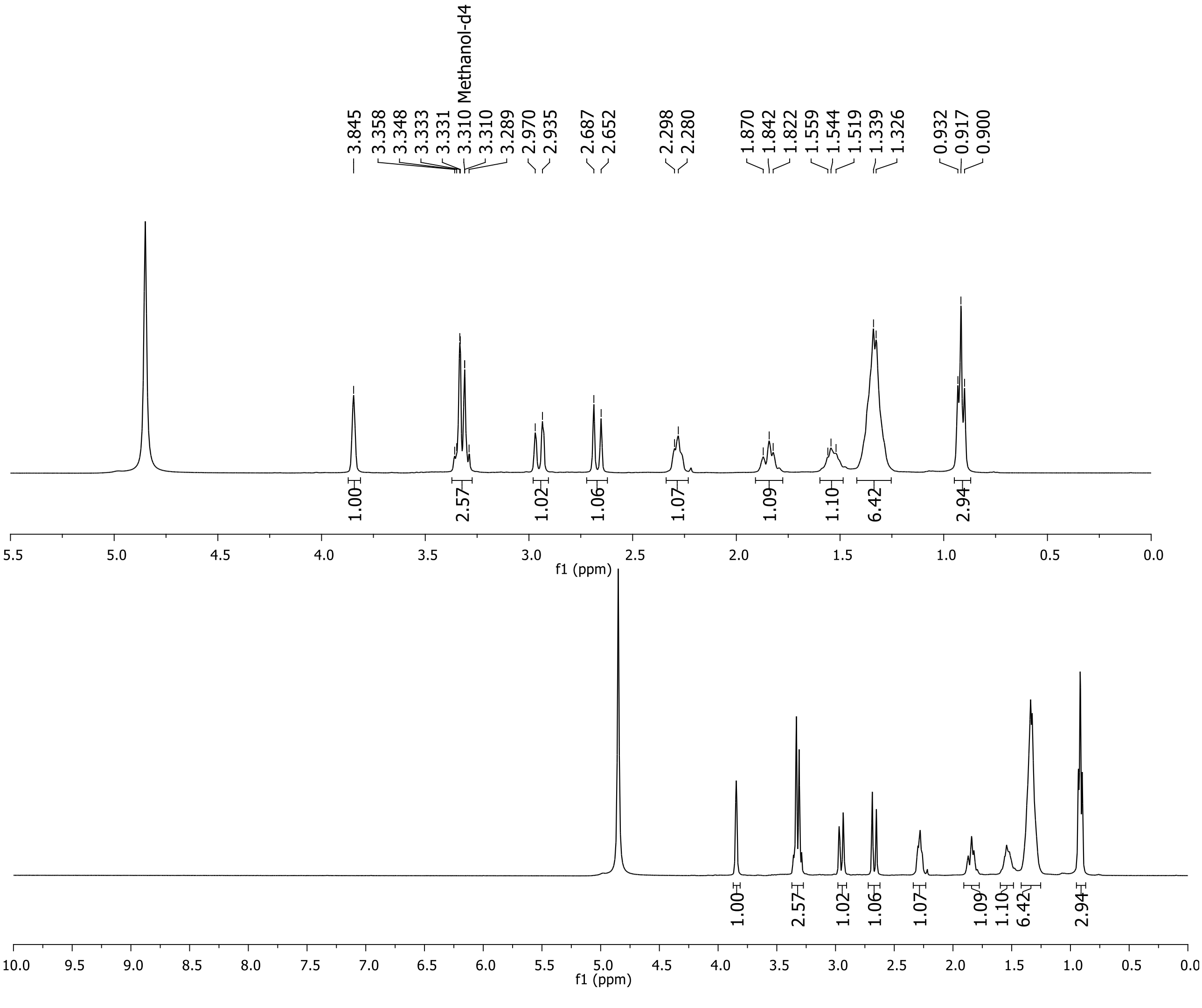

Figure S15. ^{1}H-NMR spectrum of compound **12** (400 MHz, CD_3OD).

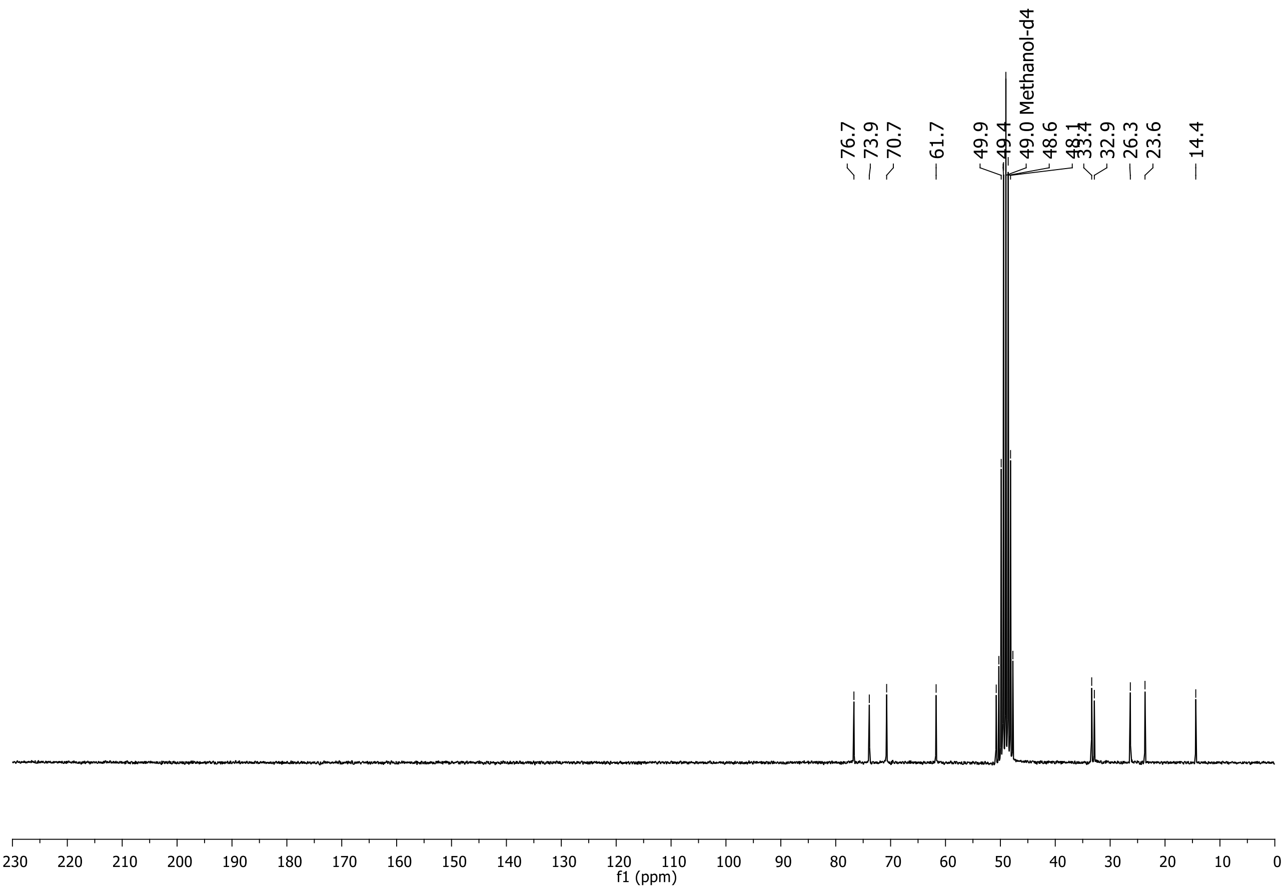

Figure S16. ^{13}C-NMR spectrum of compound **12** (50 MHz, CD_3OD).

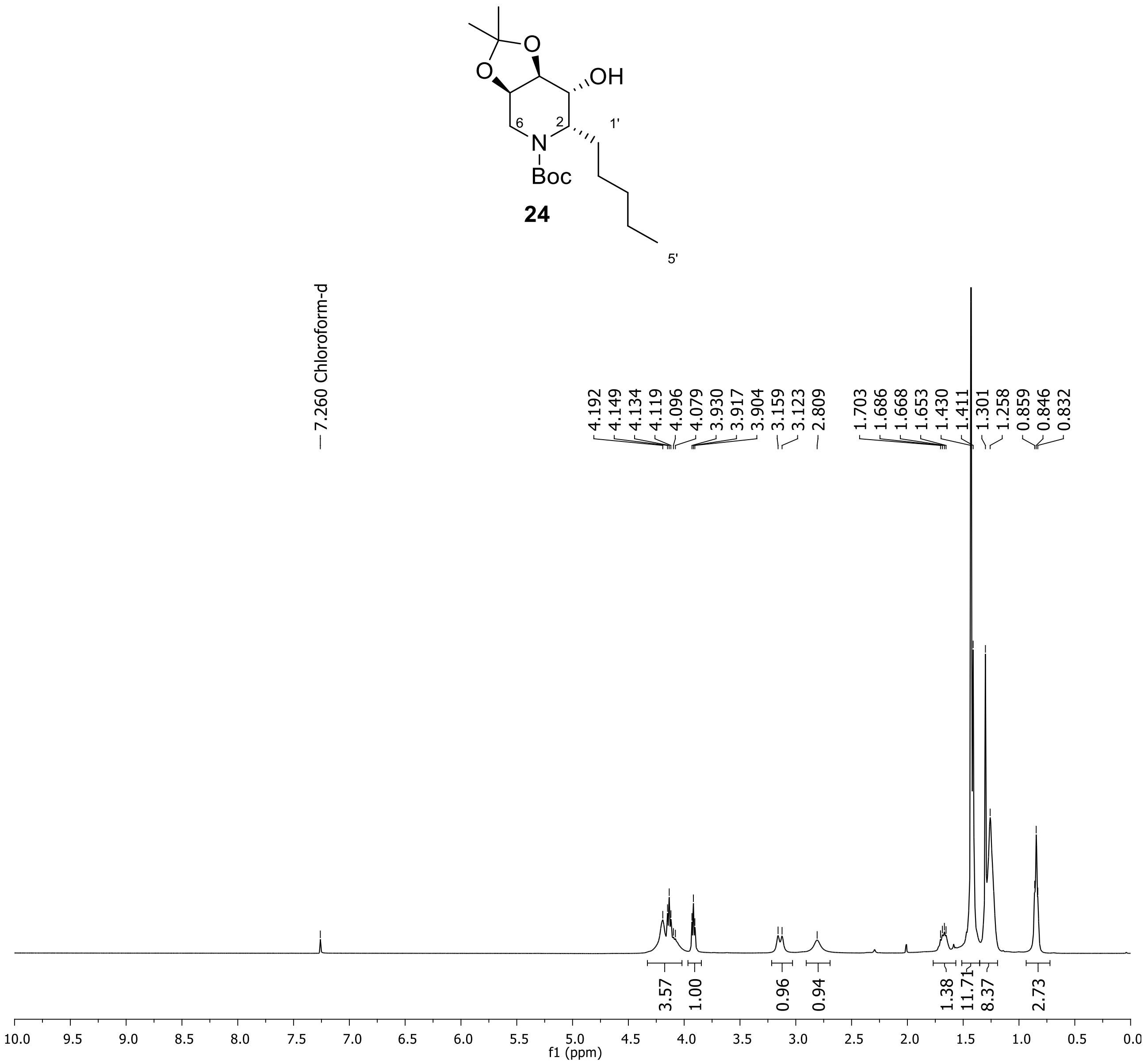

Figure S17. ^{1}H-NMR spectrum of compound **24** (400 MHz, $CDCl_3$).

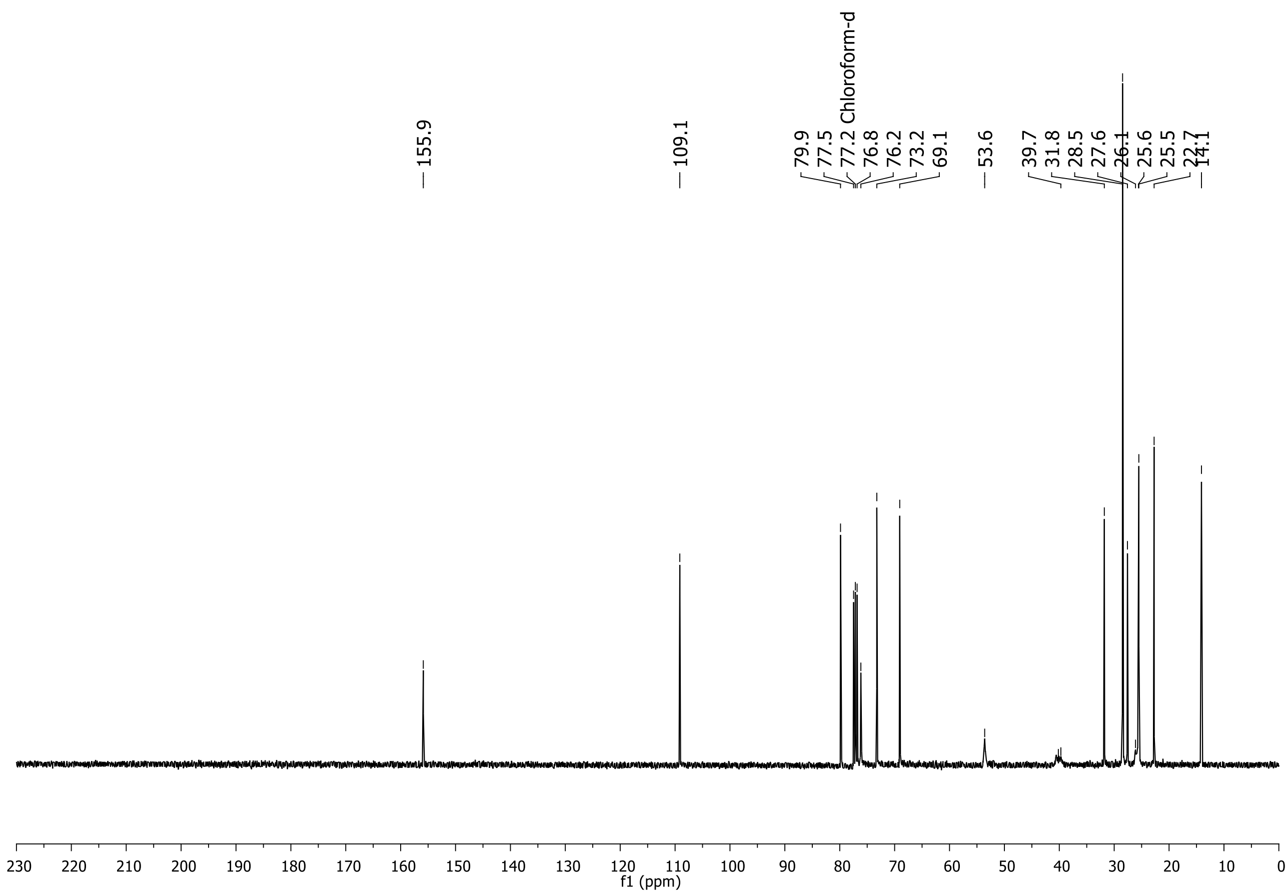

Figure S18. ^{13}C-NMR spectrum of compound **24** (100 MHz, $CDCl_3$).

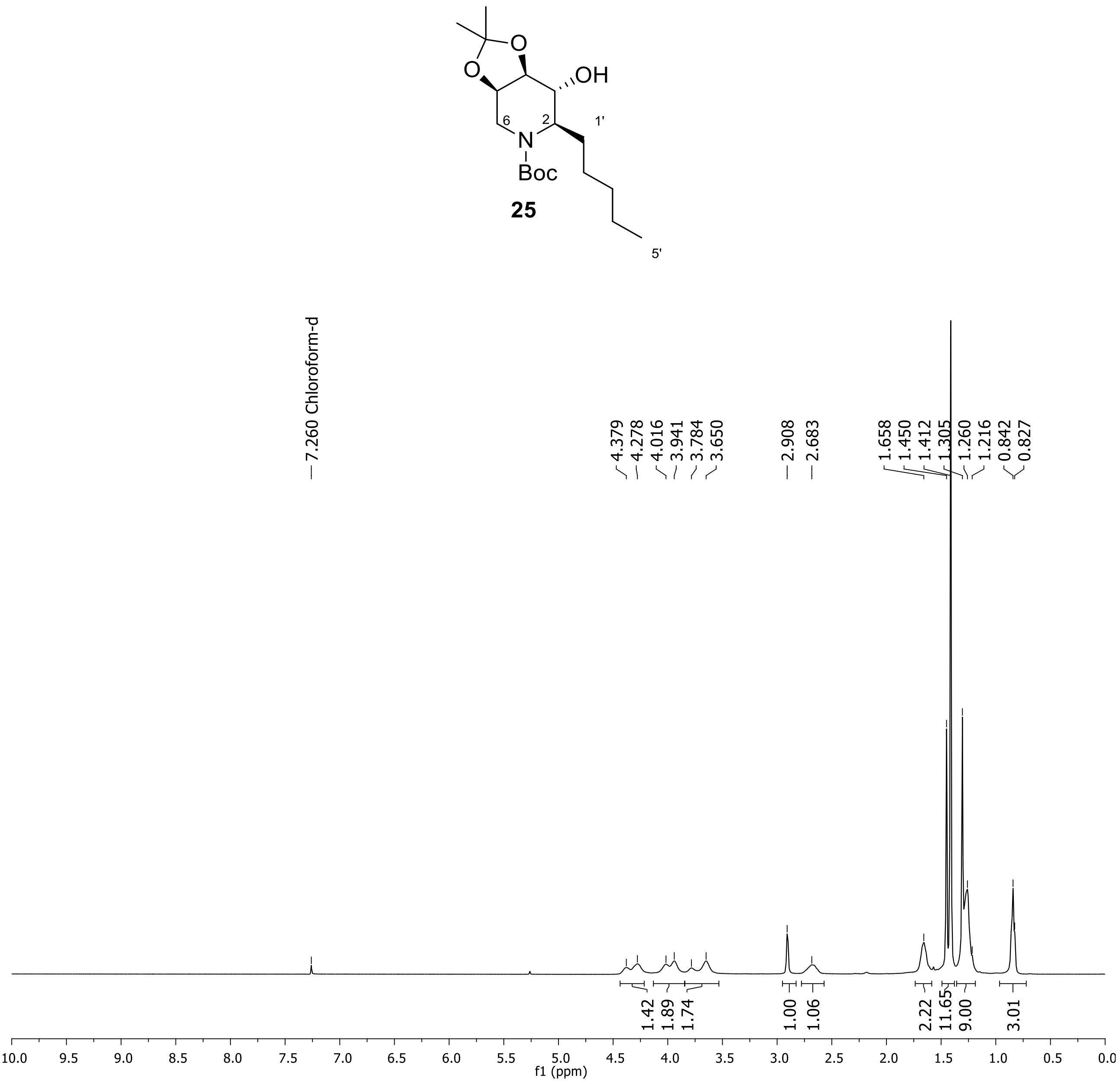

Figure S19. ^{1}H-NMR spectrum of compound **25** (400 MHz, $CDCl_3$).

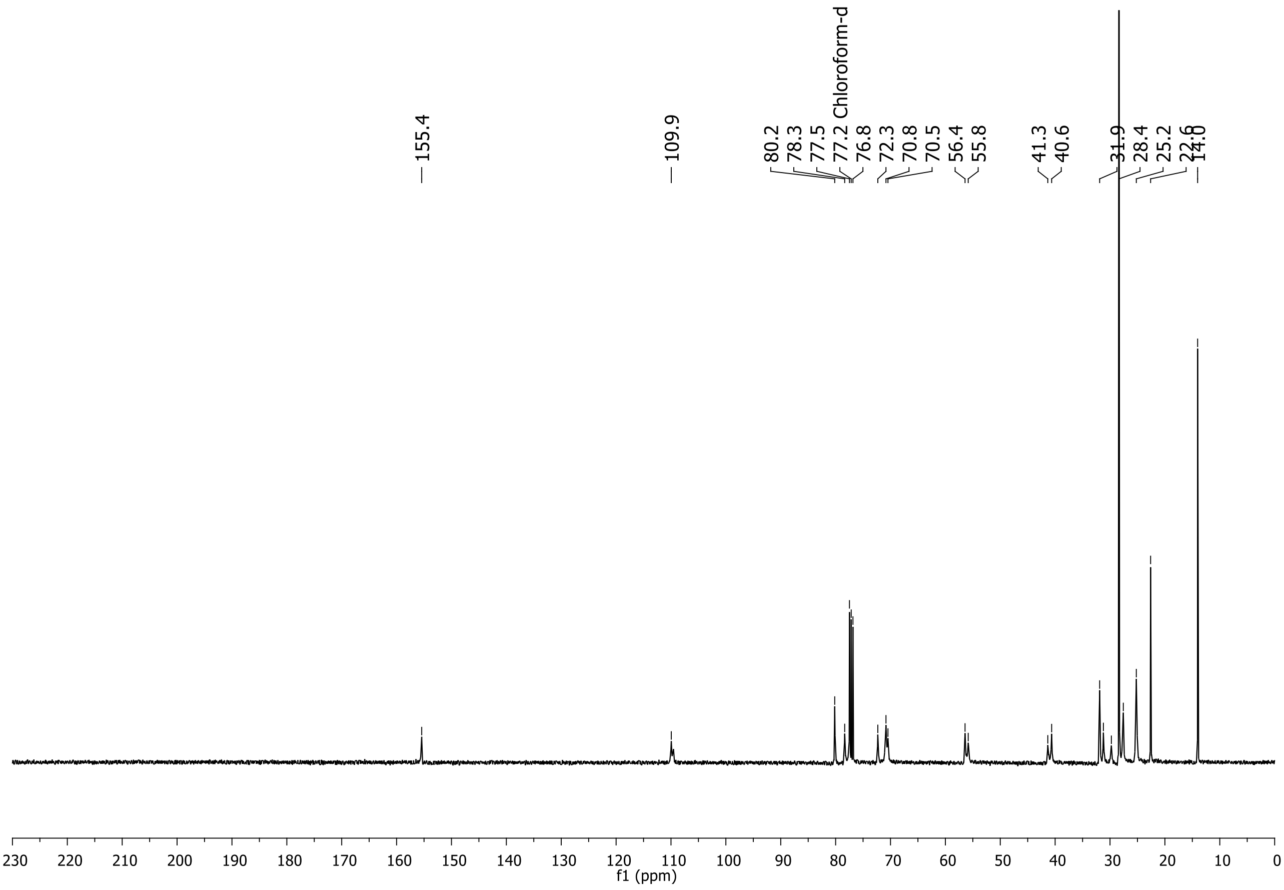

Figure S20. ^{13}C-NMR spectrum of compound **25** (50 MHz, $CDCl_3$).

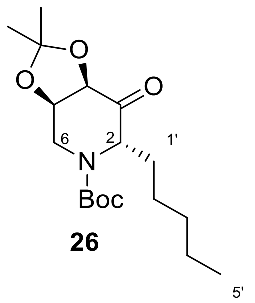

26

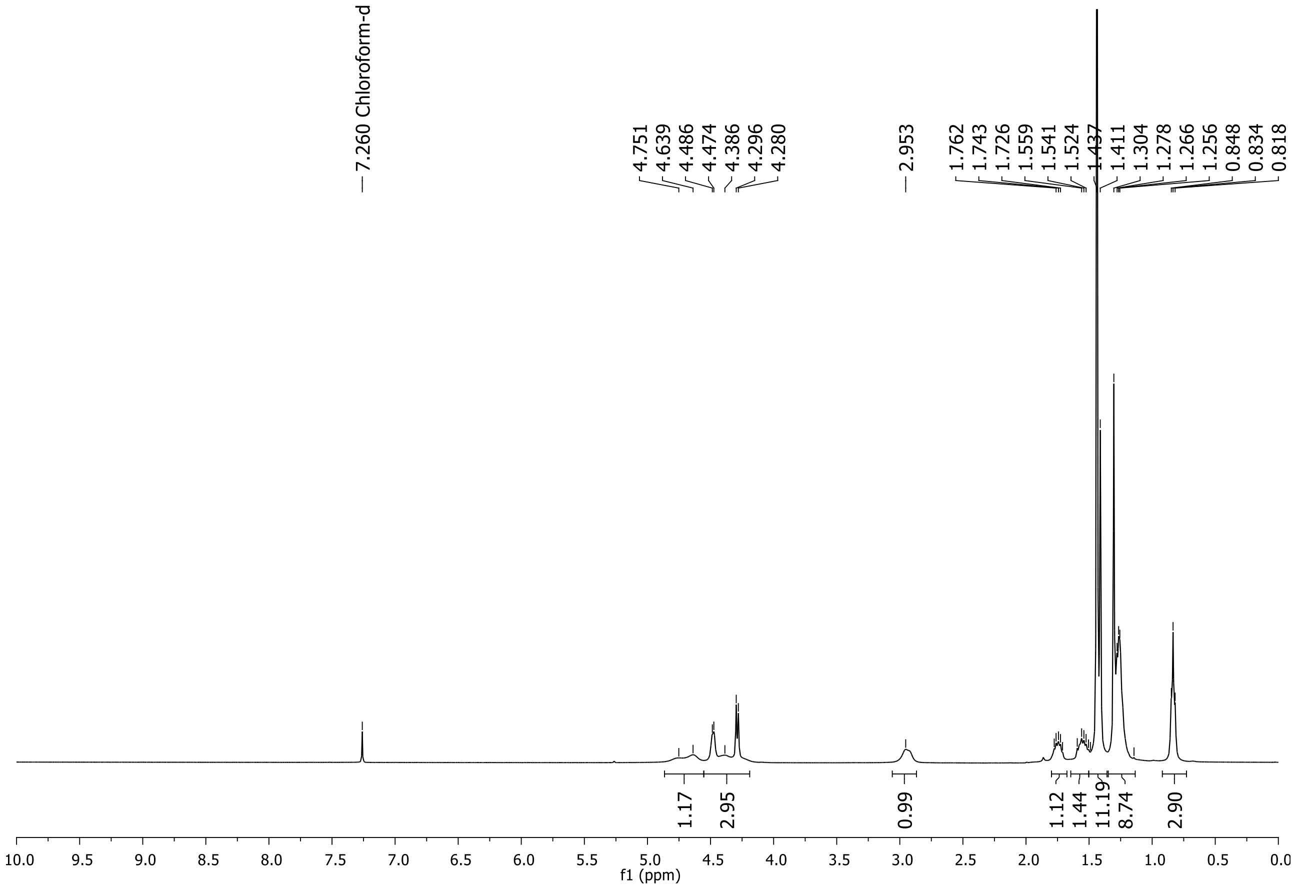

Figure S21. ^{1}H-NMR spectrum of compound **26** (400 MHz, $CDCl_3$).

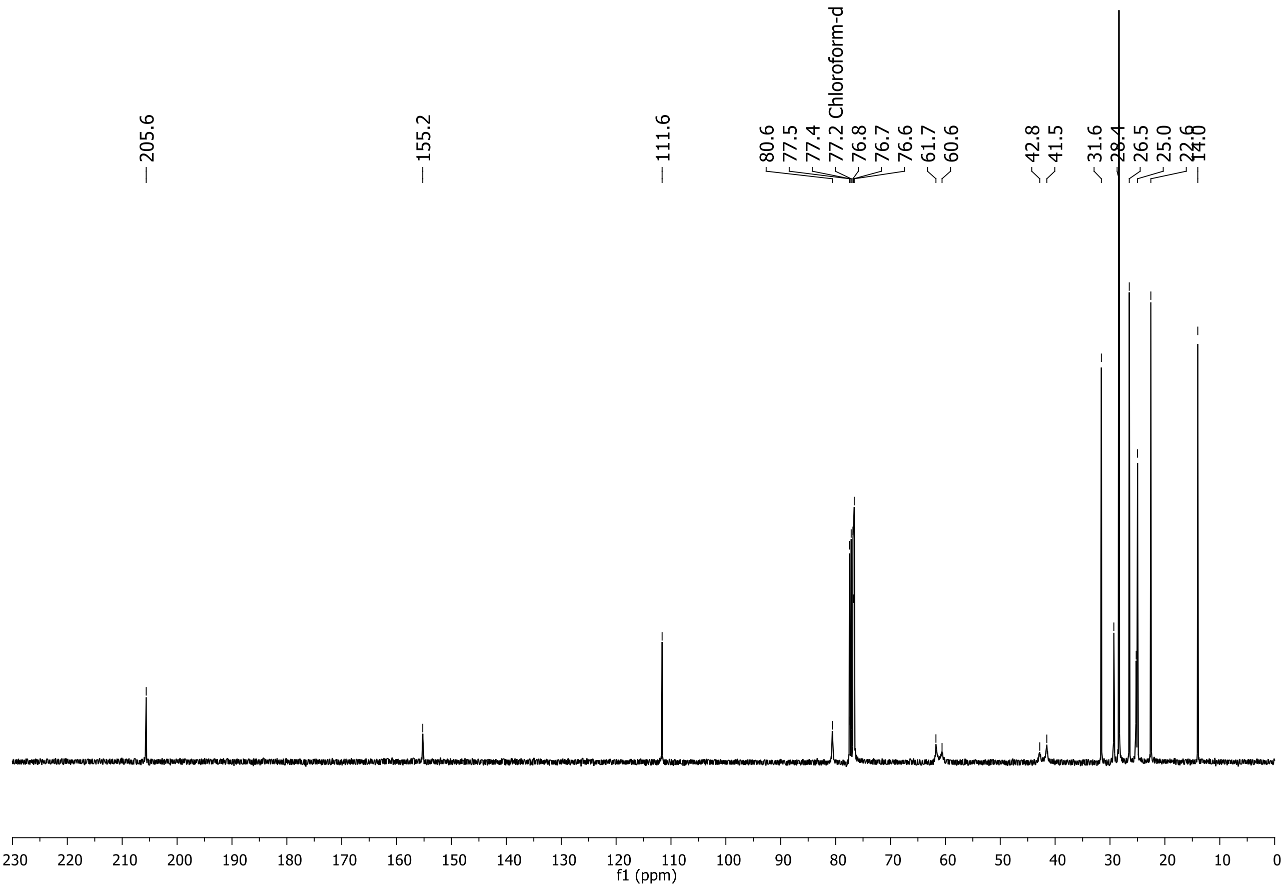

Figure S22. ^{13}C-NMR spectrum of compound **26** (100 MHz, $CDCl_3$).

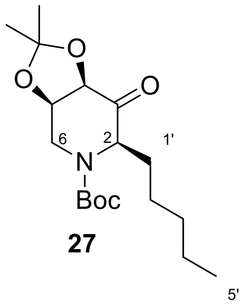

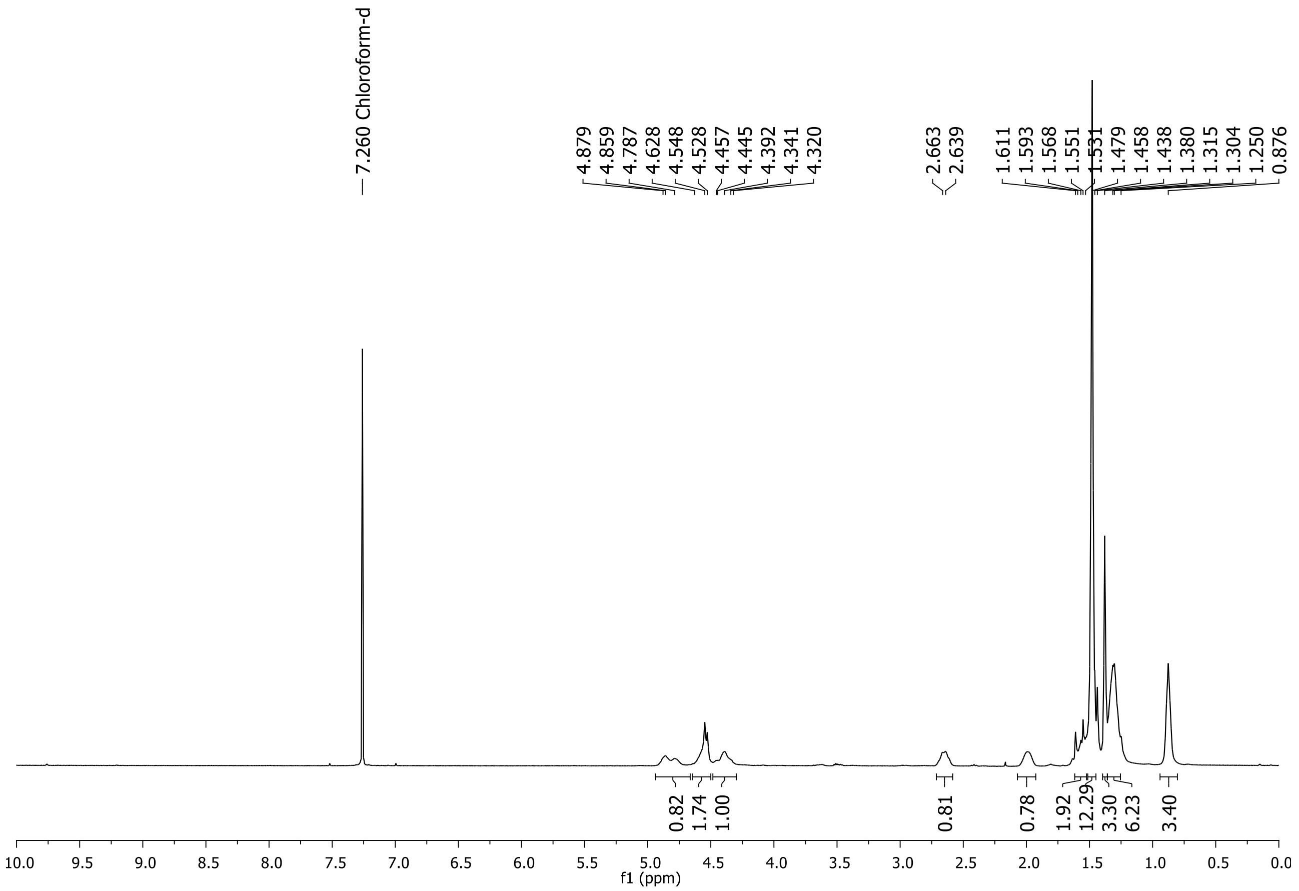

Figure S23. ^{1}H-NMR spectrum of compound **27** (400 MHz, $CDCl_3$).

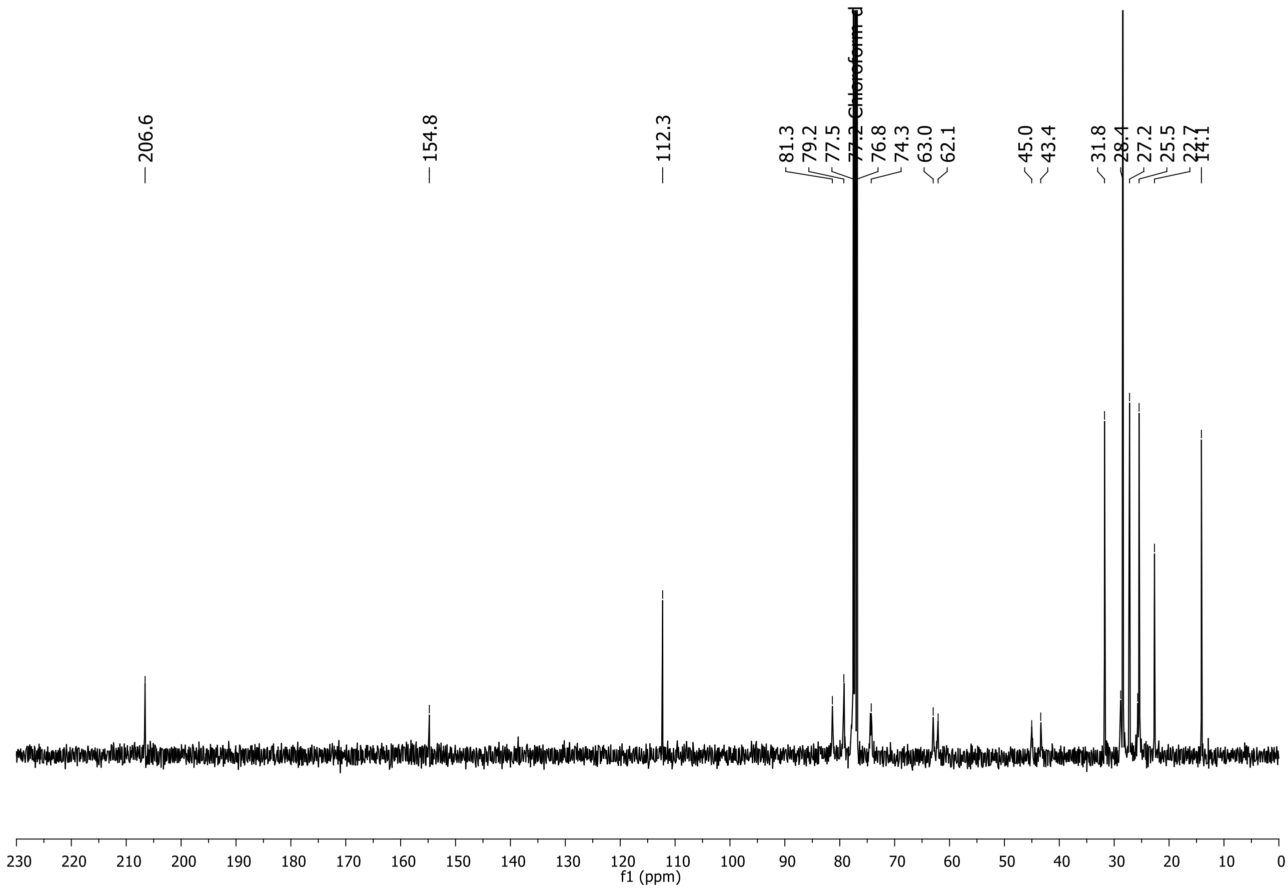

Figure S24. ^{13}C-NMR spectrum of compound **27** (50 MHz, $CDCl_3$).

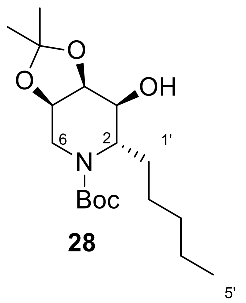

28

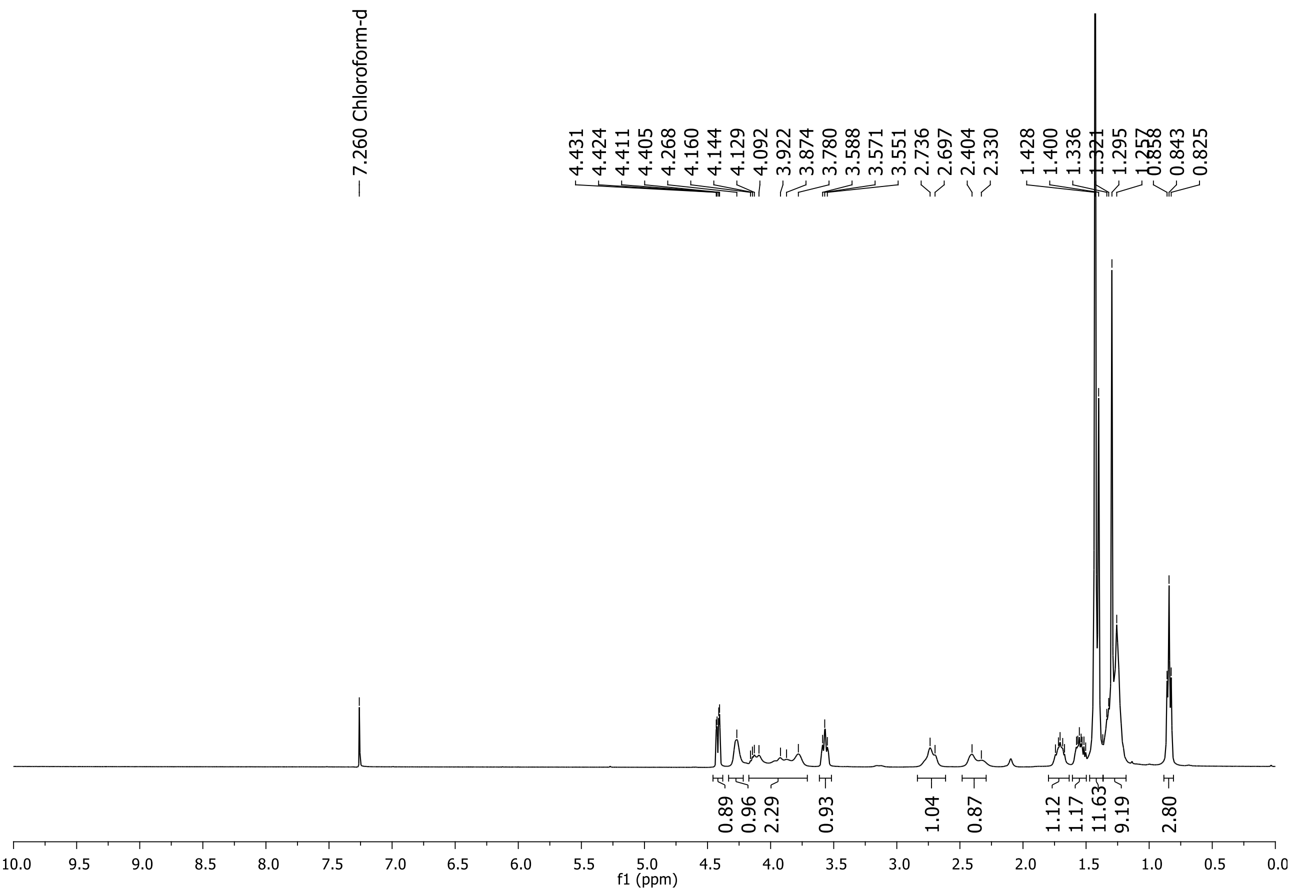

Figure S25. ^{1}H-NMR spectrum of compound **28** (400 MHz, $CDCl_3$).

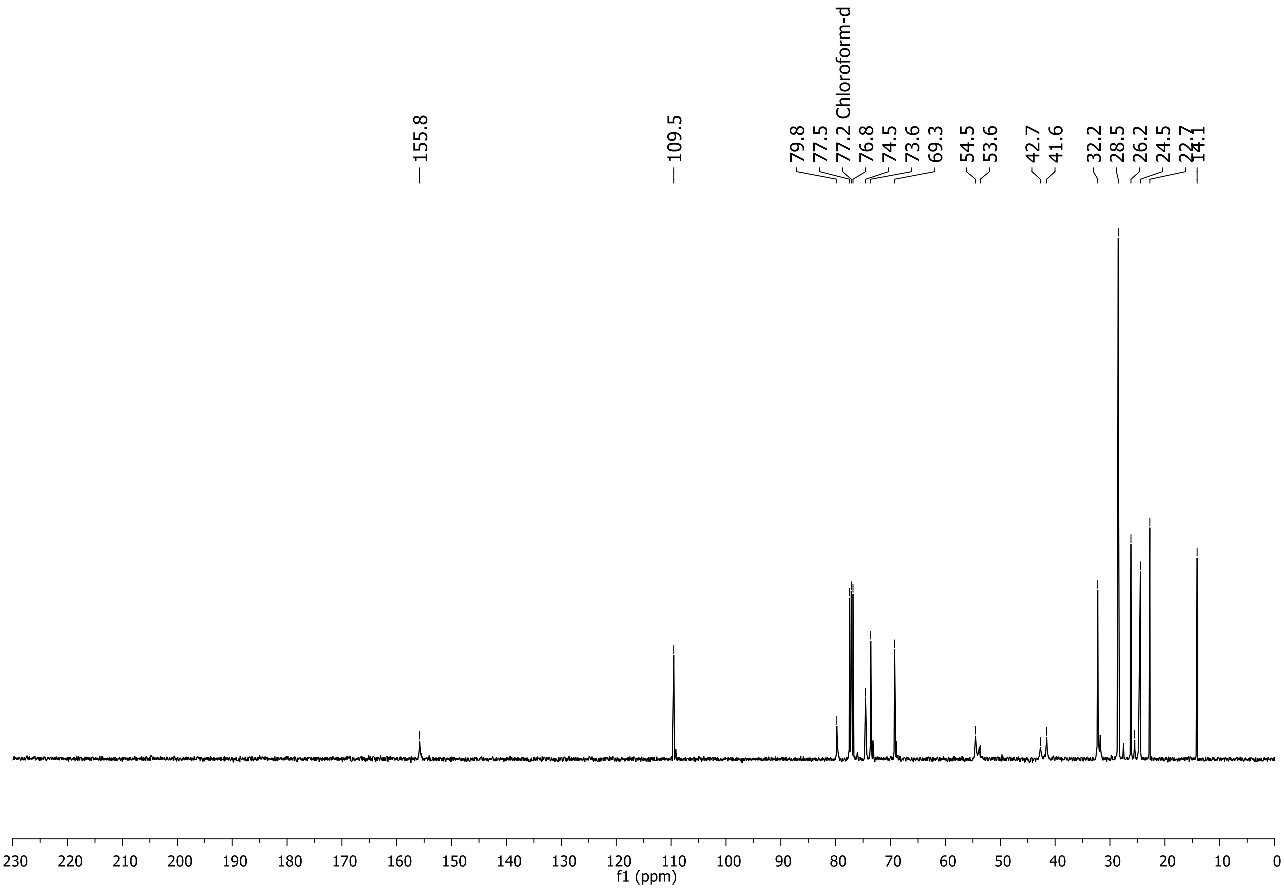

Figure S26. ^{13}C-NMR spectrum of compound **28** (100 MHz, $CDCl_3$).

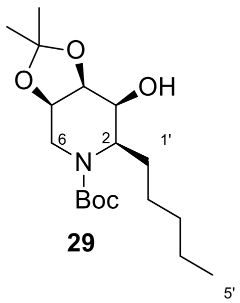

29

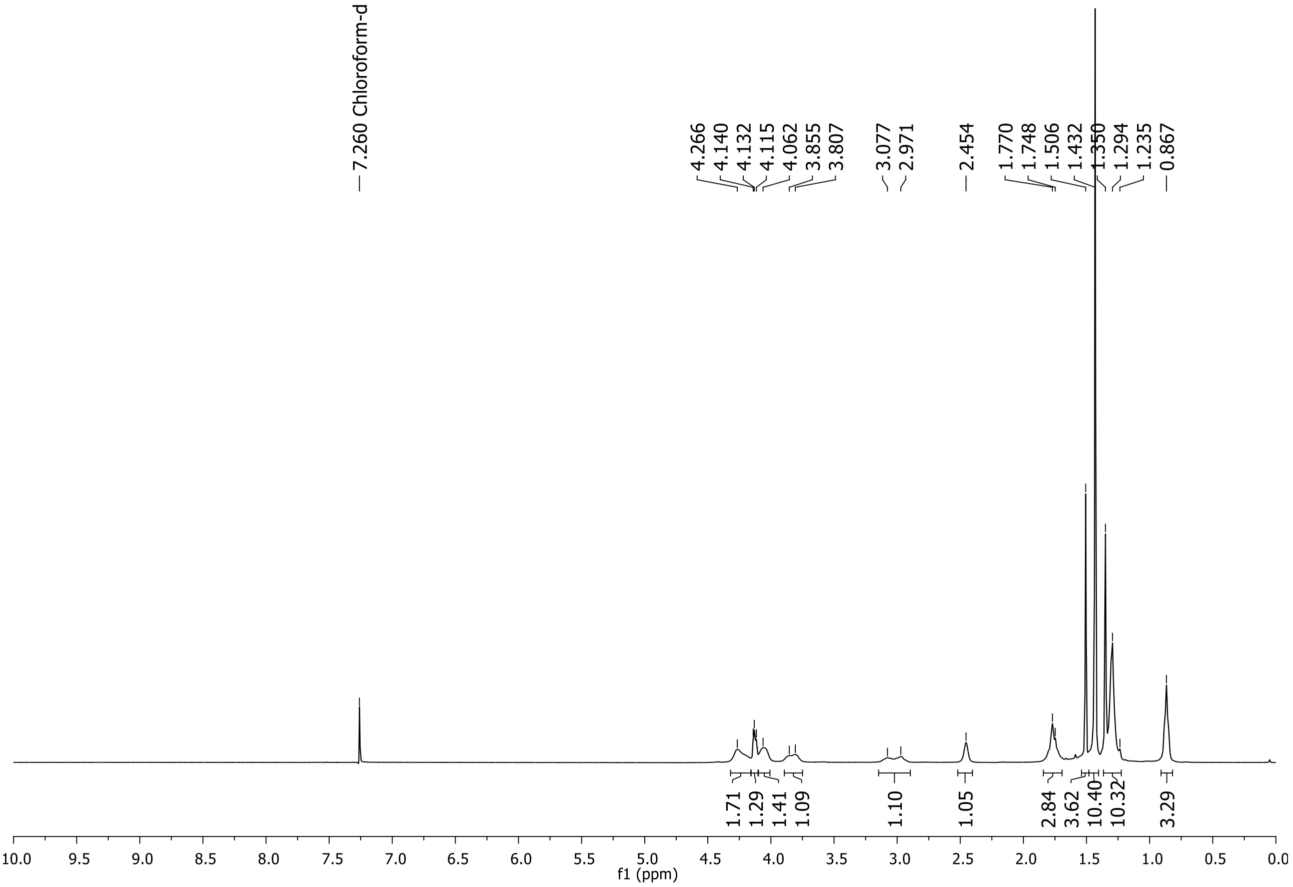

Figure S27. ^{1}H-NMR spectrum of compound **29** (400 MHz, $CDCl_3$).

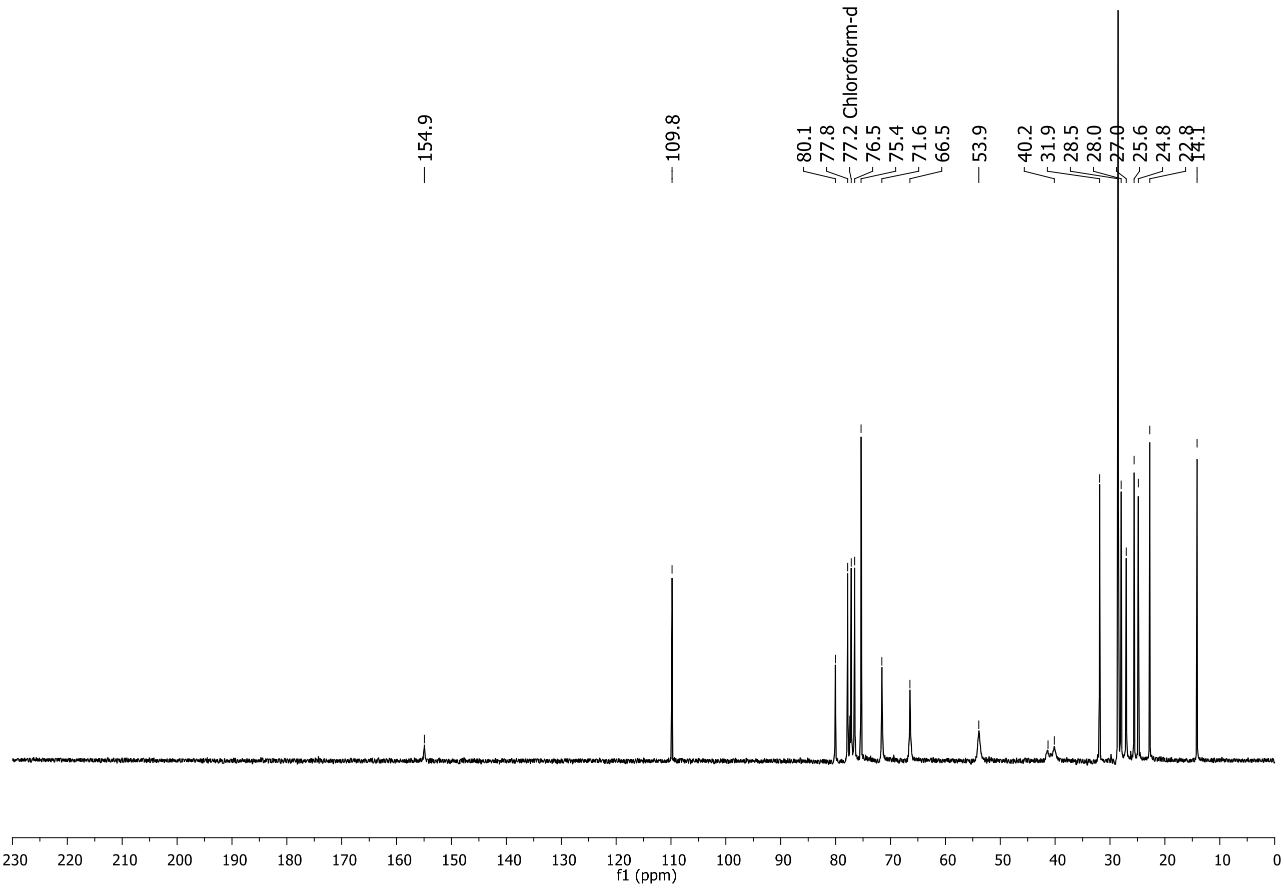

Figure S28. ^{13}C-NMR spectrum of compound **29** (100 MHz, $CDCl_3$).

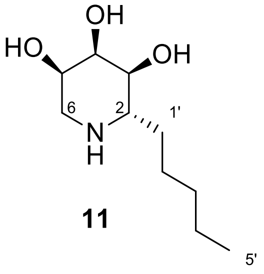

11

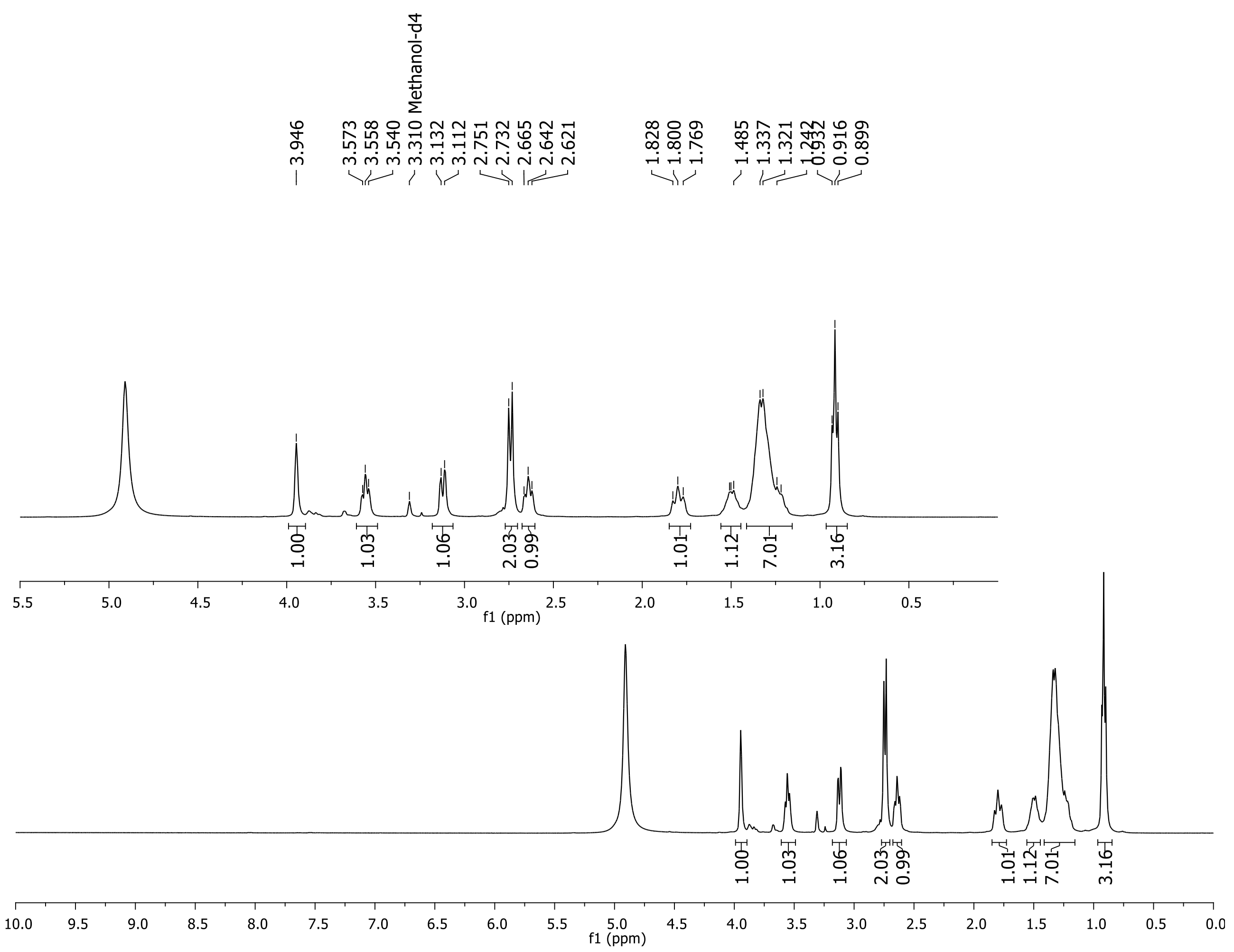

Figure S29. ^{1}H-NMR spectrum of compound **11** (400 MHz, CD_3OD).

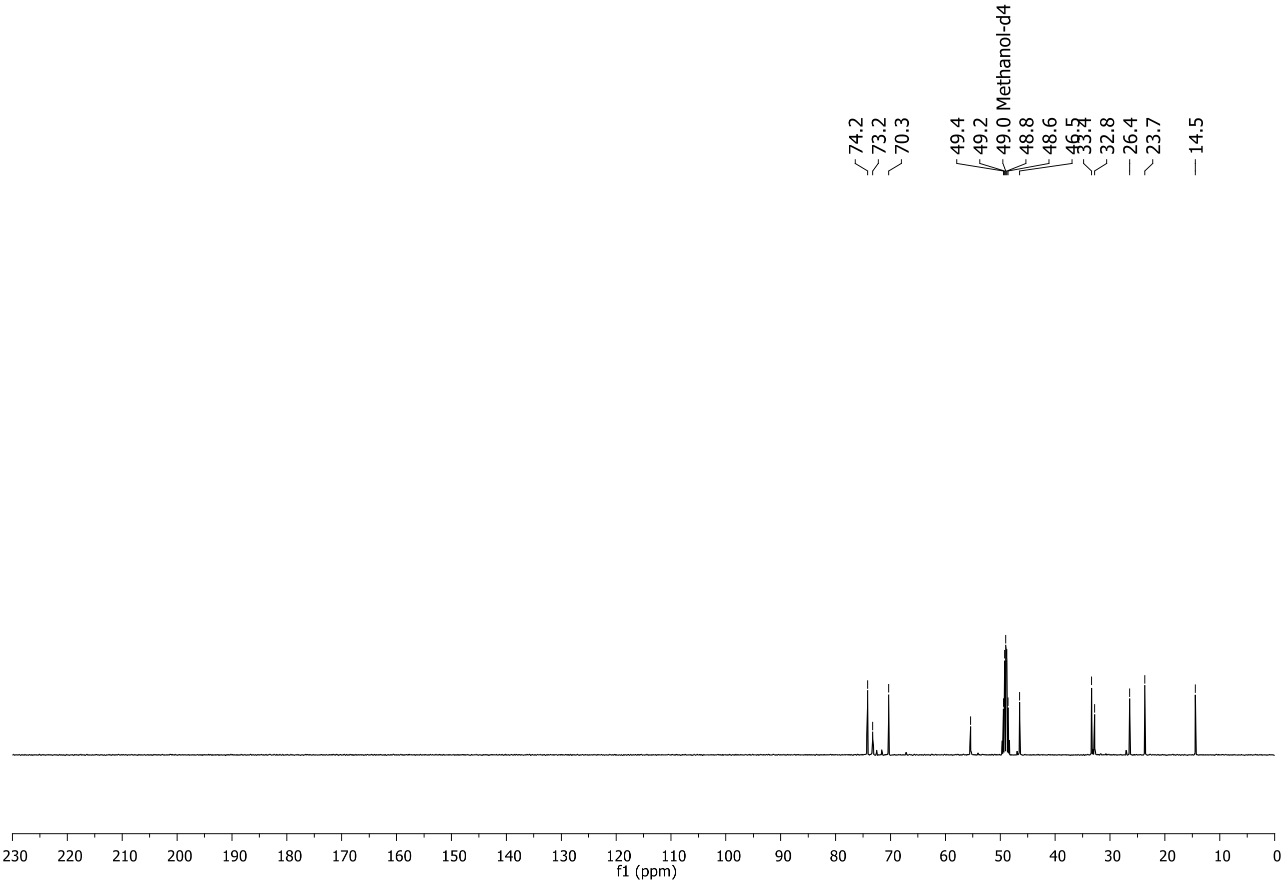

Figure S30. ^{13}C-NMR spectrum of compound **11** (100 MHz, CD_3OD).

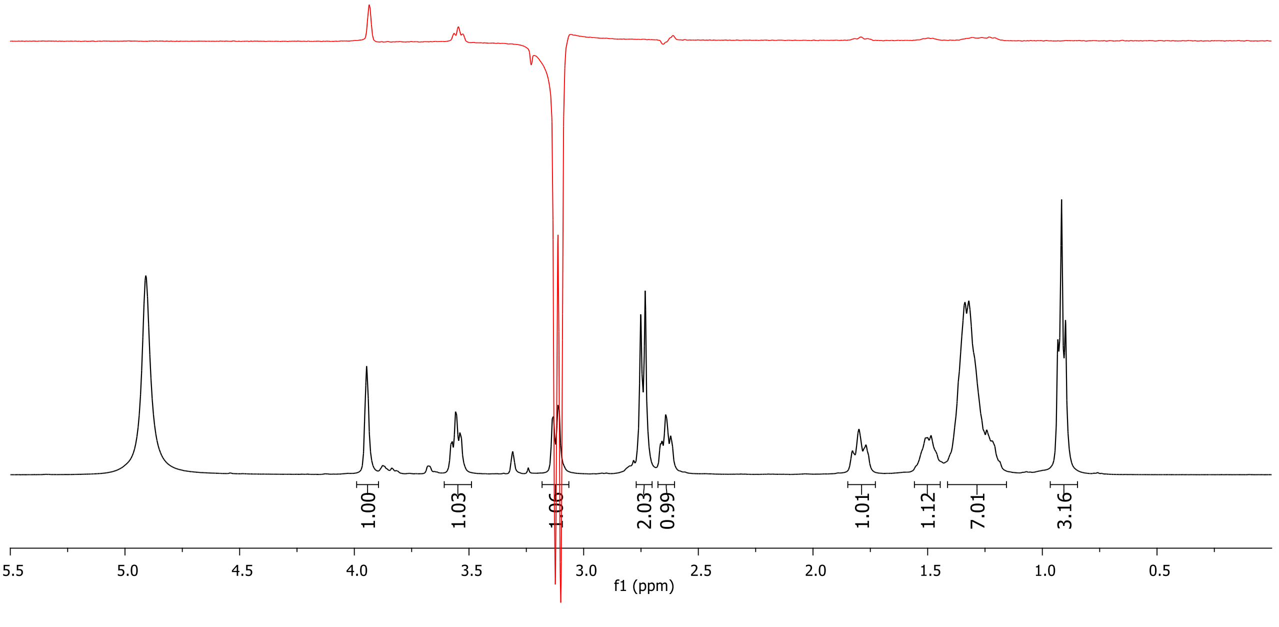

Figure S31: ^{1}D-NOESY spectra performed on compound **11**. Irradiation of H-3 gave a NOE at H-5.

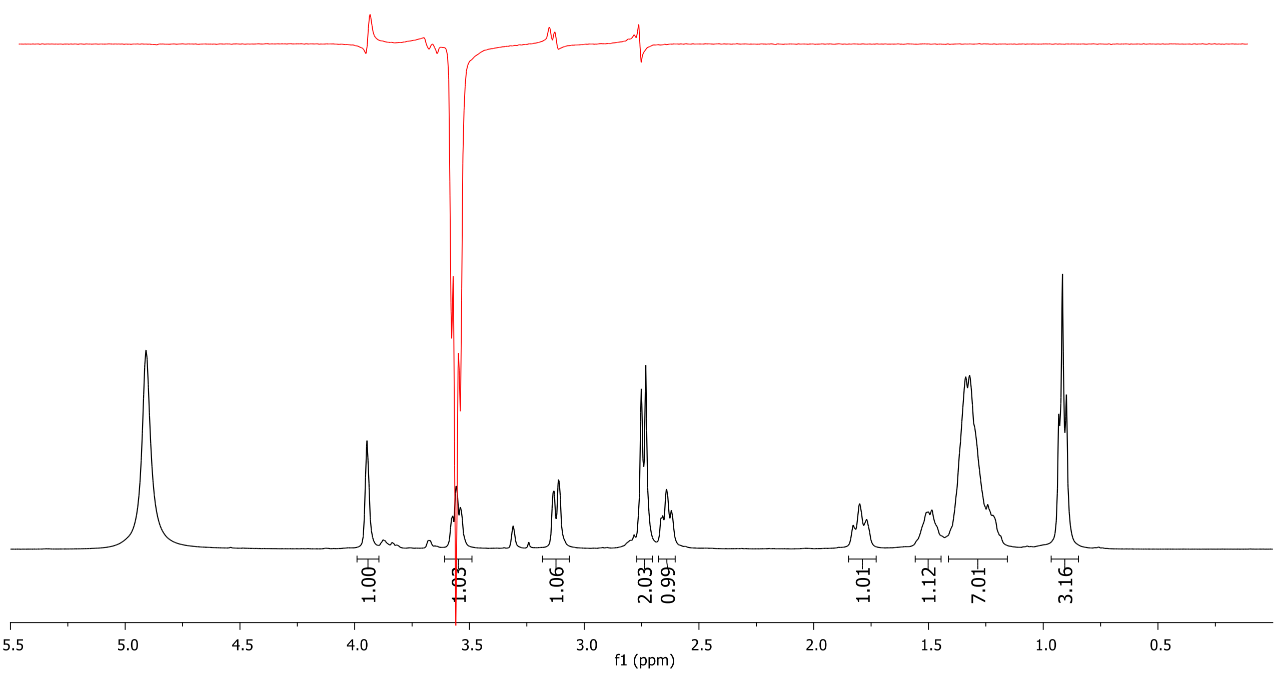

Figure S32: [1]D-NOESY spectra performed on compound **11**. Irradiation of H-5 gave a NOE at H-3.

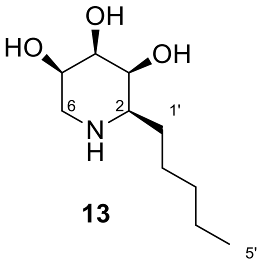

13

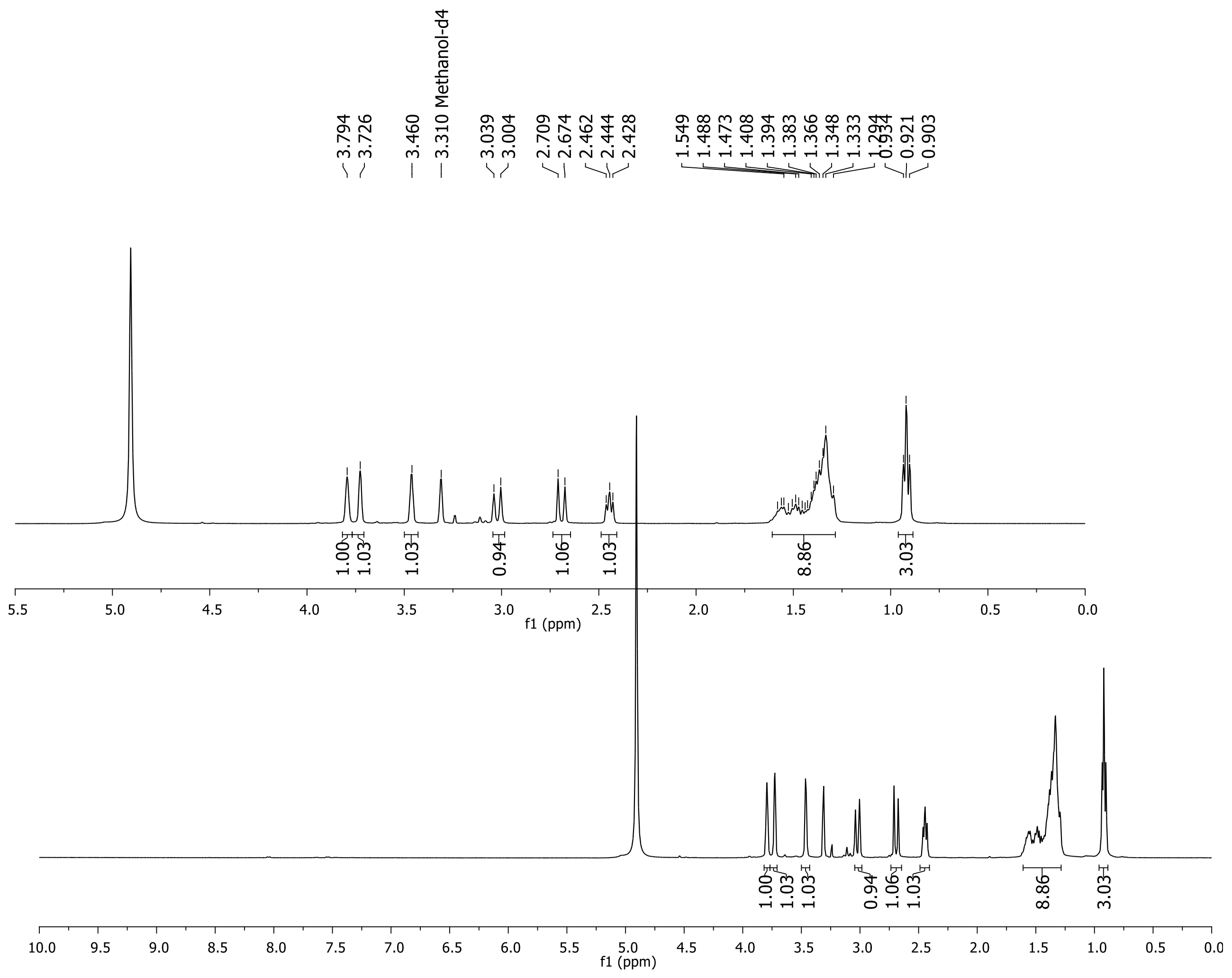

Figure S33. ^{1}H-NMR spectrum of compound **13** (400 MHz, CD_3OD).

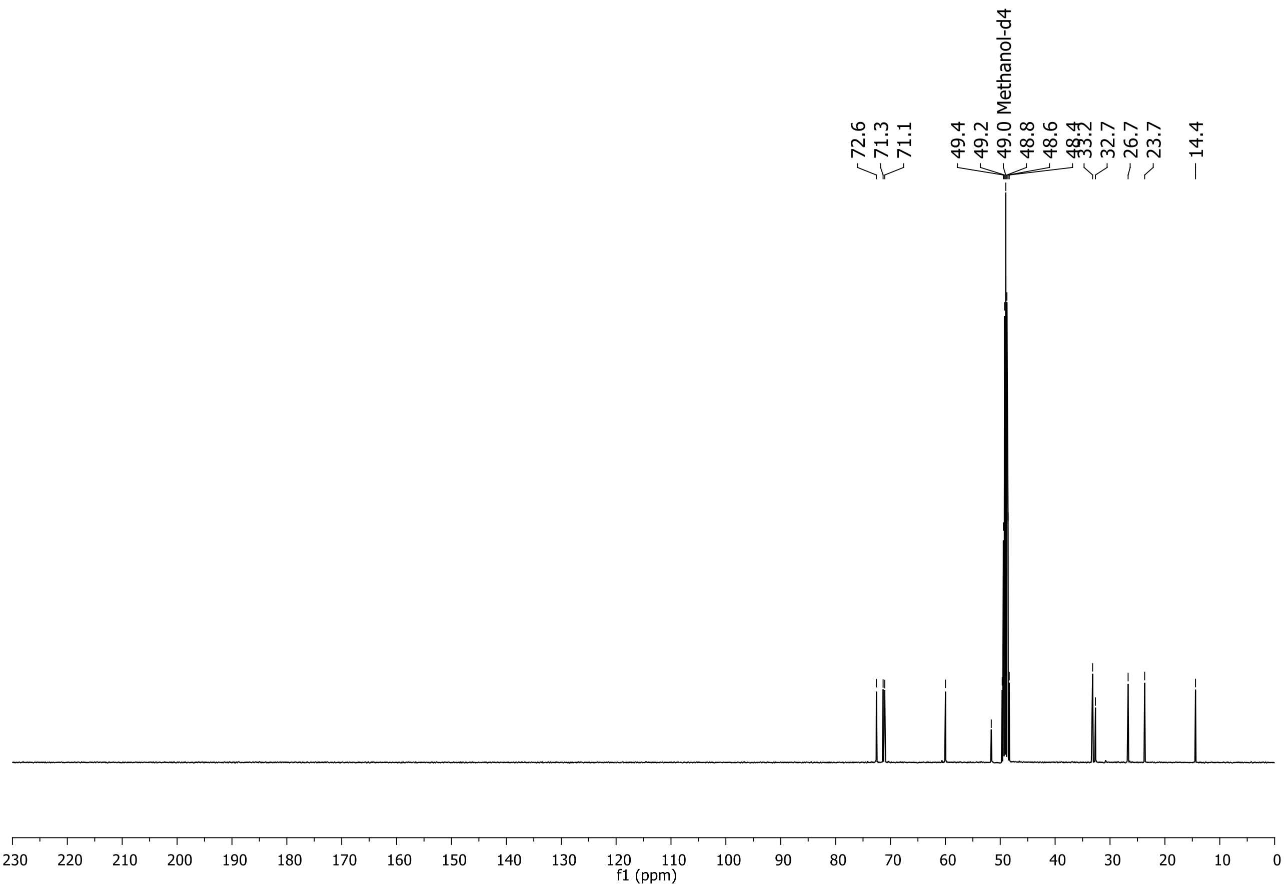

Figure S34. ^{13}C-NMR spectrum of compound **13** (50 MHz, CD_3OD).

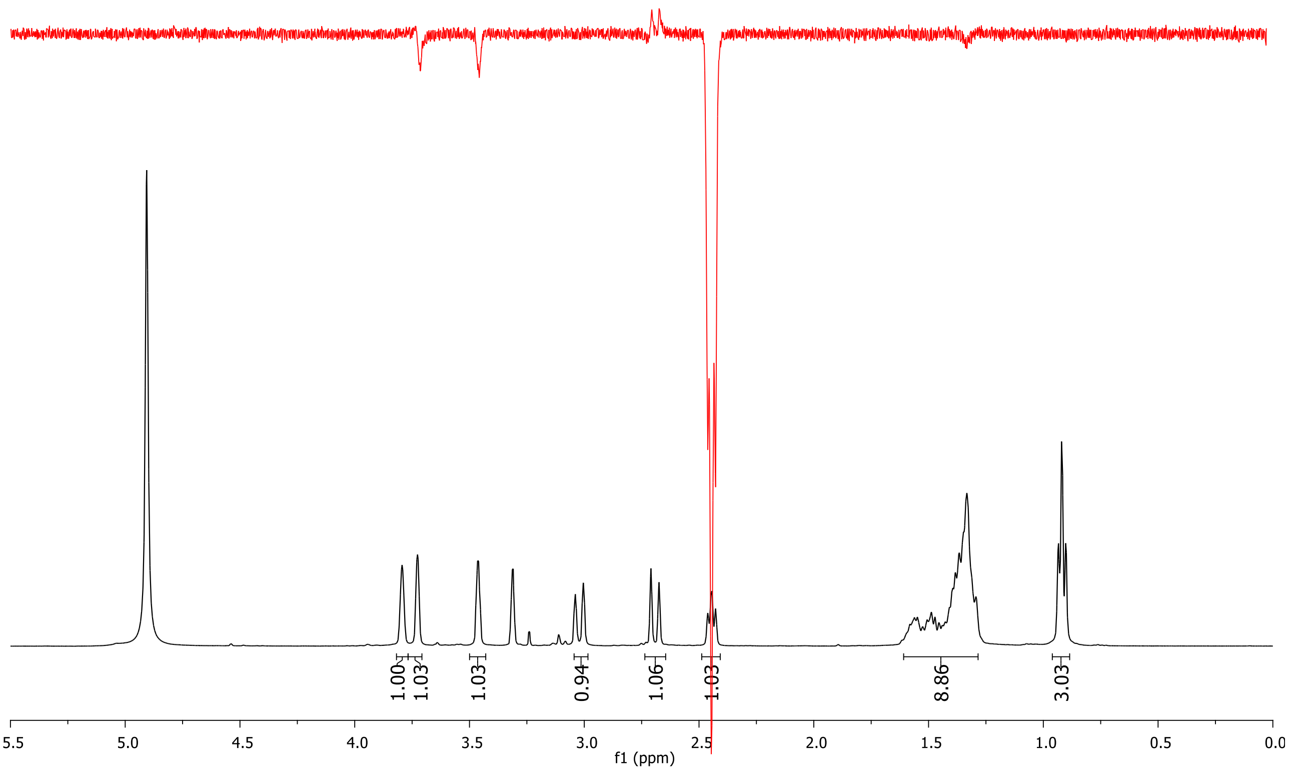

Figure S35: [1]D-NOESY spectra performed on compound **13**. Irradiation of H-2 gave a NOE at H-4 and H_b-6.

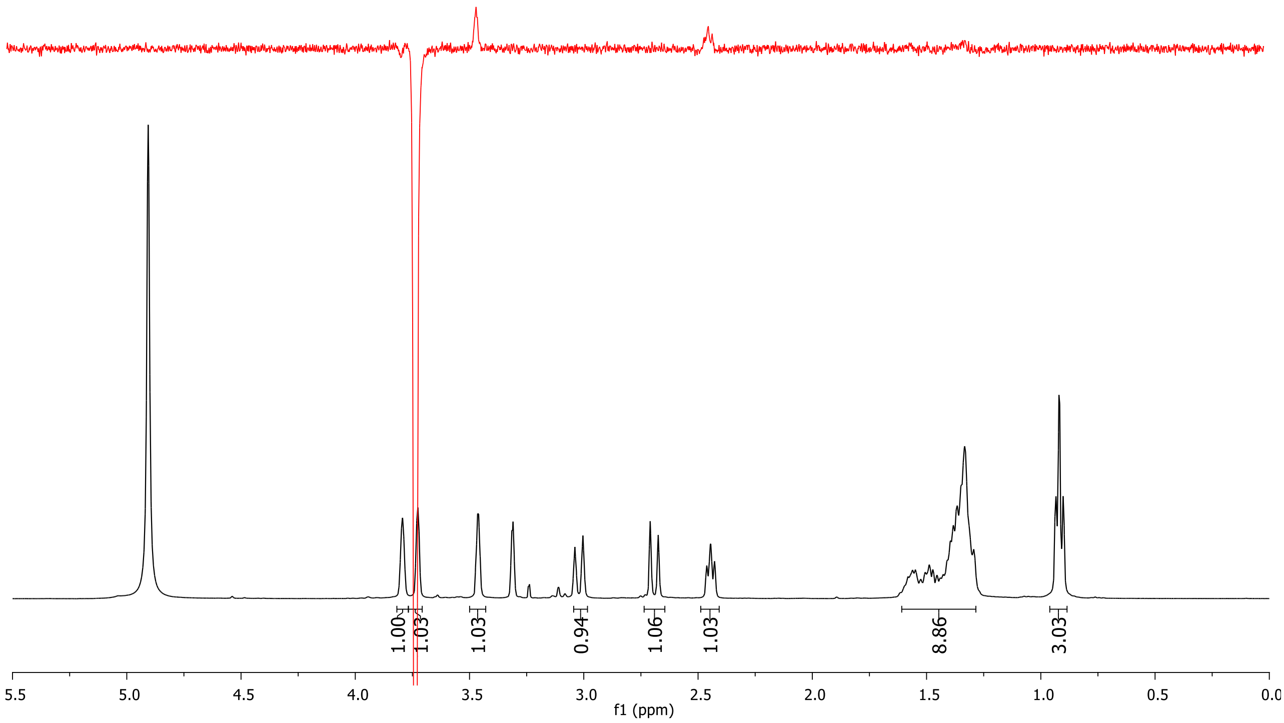

Figure S36: ^{1}D-NOESY spectra performed on compound **13**. Irradiation of H-4 gave a NOE at H-2 and H_b-6.

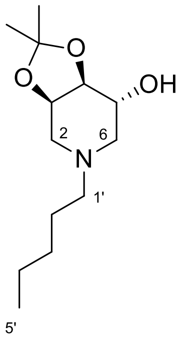

30

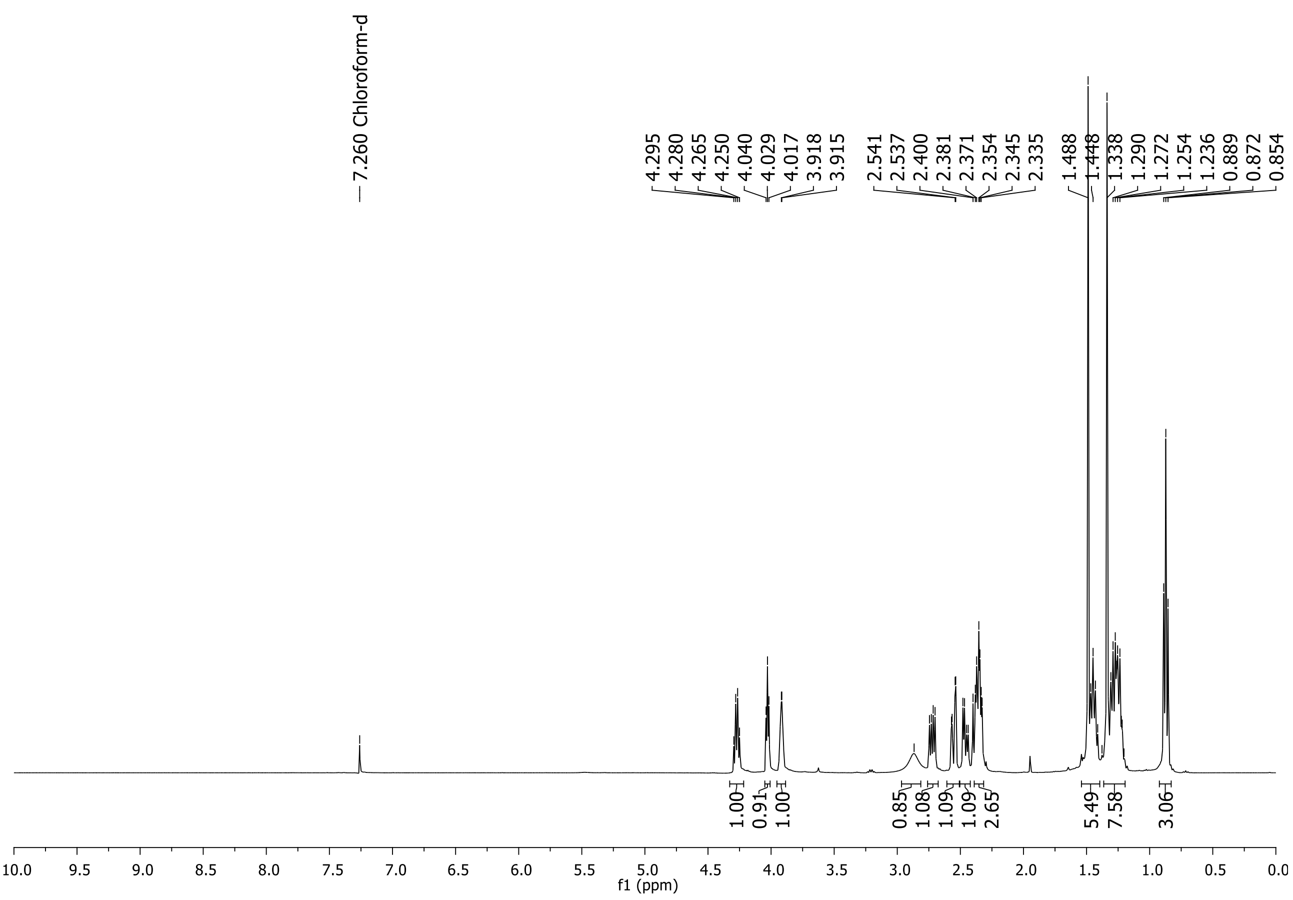

Figure S37. ^{1}H-NMR spectrum of compound **30** (400 MHz, $CDCl_3$).

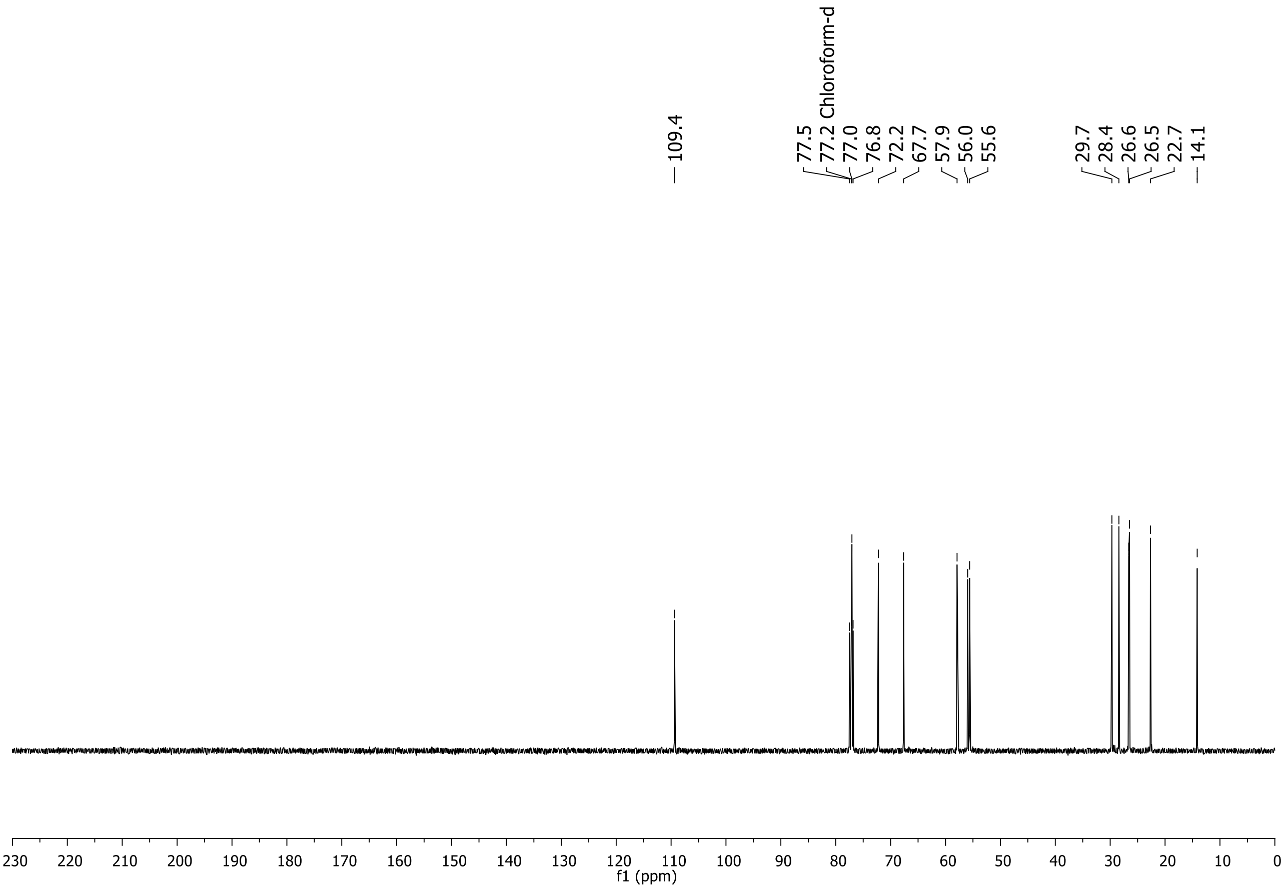

Figure S38. ^{13}C-NMR spectrum of compound **30** (100 MHz, $CDCl_3$).

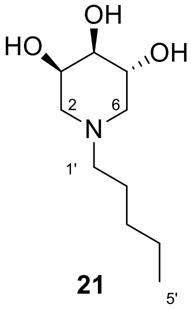

21

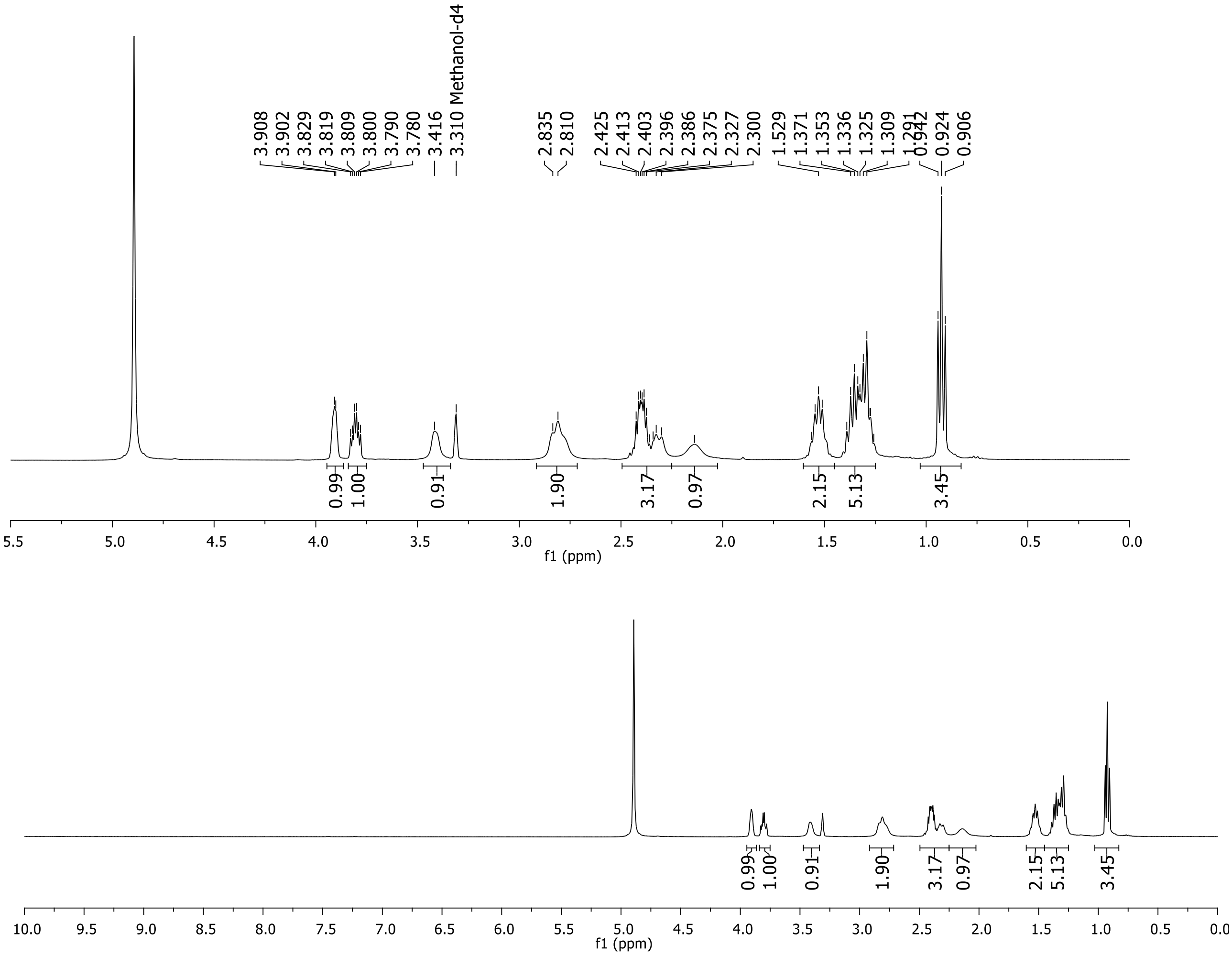

Figure S39. ^{1}H-NMR spectrum of compound **21** (400 MHz, CD_3OD).

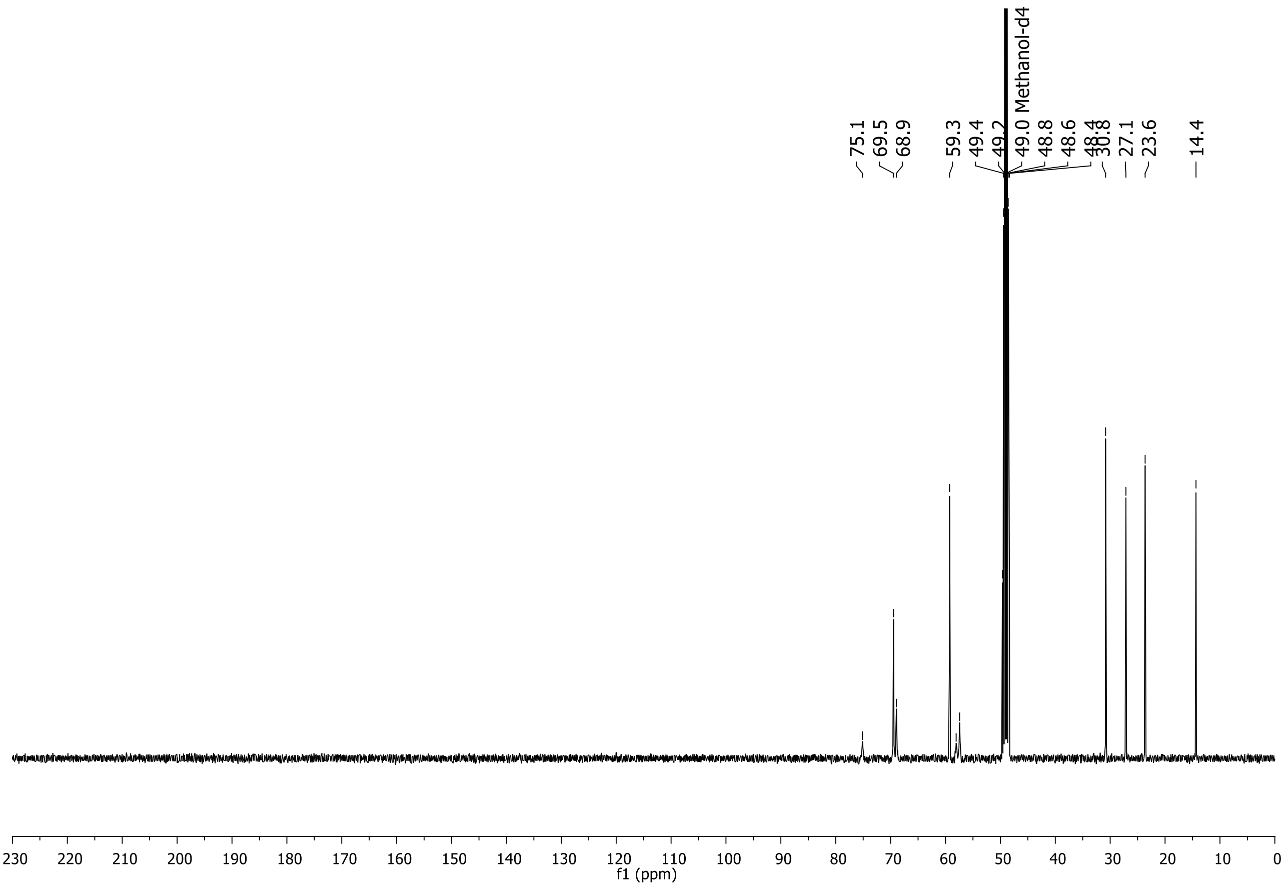

Figure S40. ^{13}C-NMR spectrum of compound **21** (400 MHz, CD_3OD).

Biological Screening towards Human Lysosomal β-Galactosidase (β-Gal) and β-Glucosidase (GCase)

- Human lysosomal β-galactosidase (β-Gal) activity

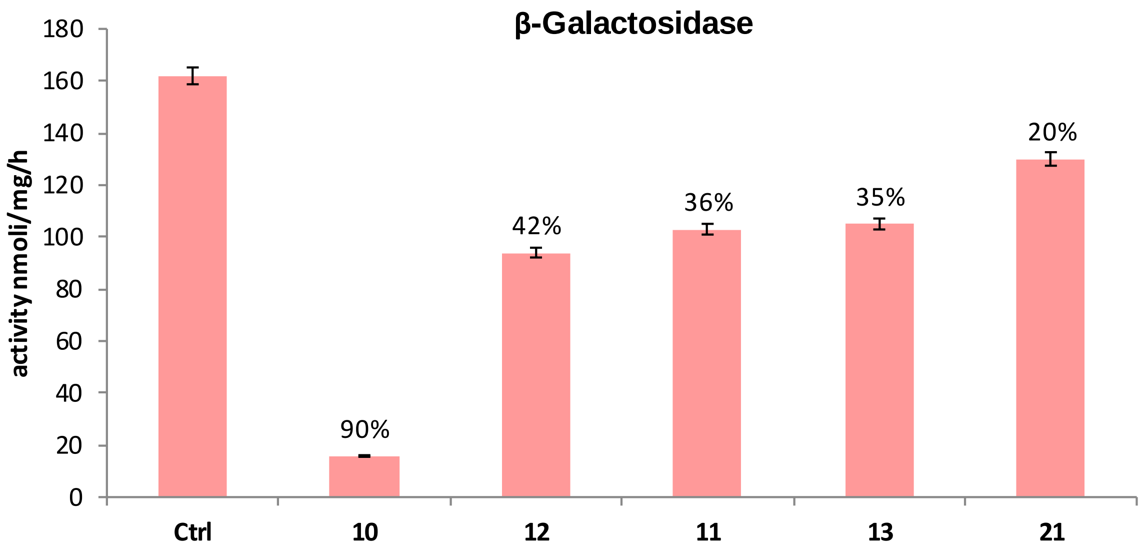

Figure S41. Activity of β-Gal in the presence of compounds (1 mM). The corresponding calculated percentage of inhibition is indicated above each bar.

For compounds showing β-Gal inhibitory activity higher than 60% at 1 mM concentration, the IC_{50} values were determined by measuring the initial hydrolysis rate with 4-methylumbelliferyl β-D-galactopyranoside (1.47 mM).

Data obtained were fitted to the following equation using the OriginPro 2021 program (OriginLab Corporation, Northampton, MA, USA).

$$\frac{Vi}{Vo} = \frac{Max - Min}{1 + \left(\frac{x}{IC_{50}}\right)^{slope}} + Min$$

where V_i/V_o, represent the ratio between the activity measured in the presence of the inhibitor (V_i) and the activity of the control without the inhibitor (V_0), "x" the inhibitor concentration, Max and Min, the maximal and minimal enzymatic activity observed, respectively.

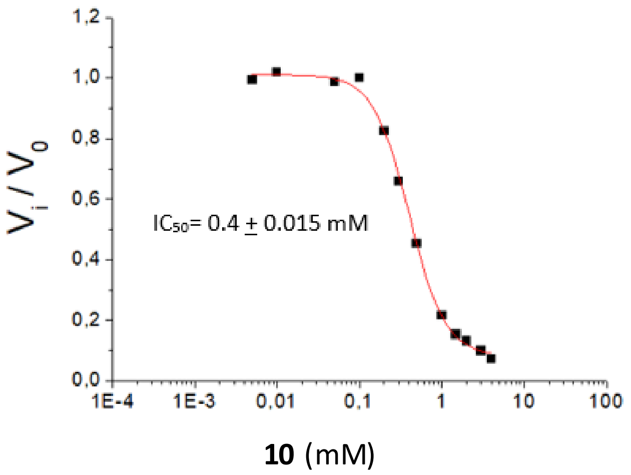

Figure S42. IC_{50} of compound **10** towards β-Gal

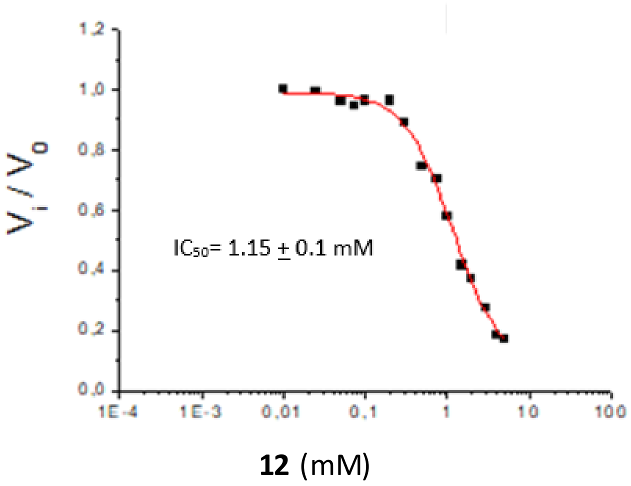

Figure S43. IC_{50} of compound **12** towards β-Gal

- Human lysosomal β-glucosidase (GCase) activity

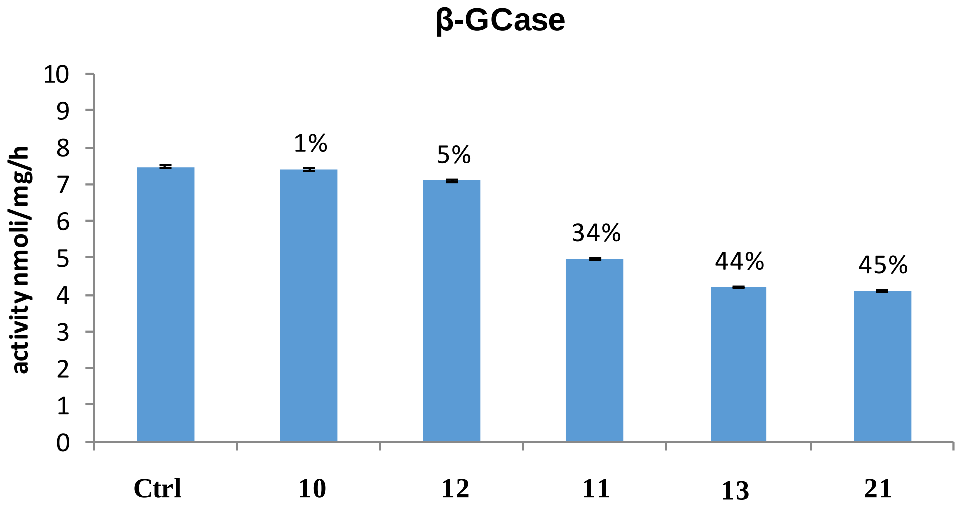

Figure S44. Activity of GCase in the presence of compounds (1 mM). The corresponding calculated percentage of inhibition is indicated above each bar.

Kinetic Analysis for compound **12** *vs* β-Gal

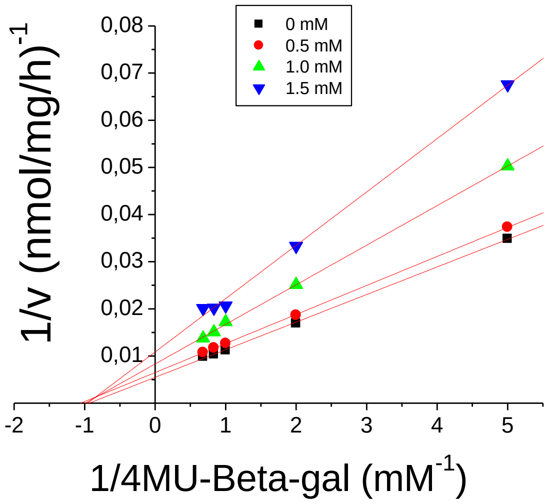

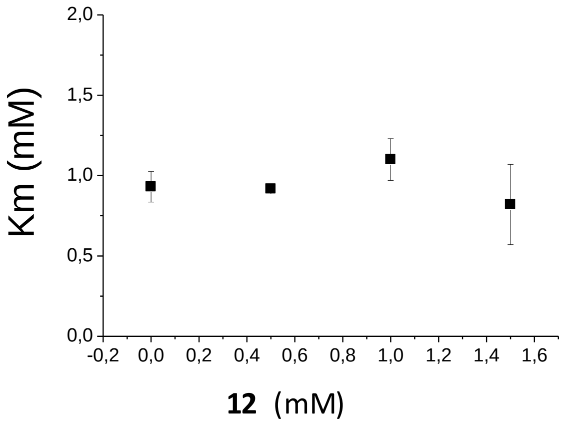

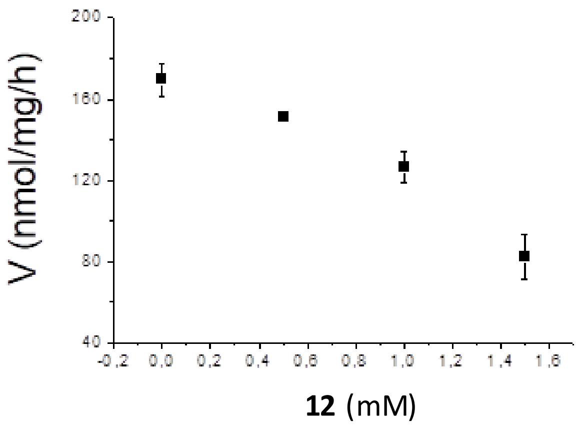

Figure S47: Kinetic analysis of compound **12** (A) Double reciprocal plots. 4-Methylumbelliferyl-β-D-glucoside was employed as a substrate. The concentrations of compound **12** are: ■, 0 mM; ●, 0.5 mM; ▲, 1.0 mM; ▼, 1.5 mM. Data reported in the figures represent the mean values — S.E.M. (n = 3). (B, C) Behavior of Km and Vmax at different concentrations of compound **12.**

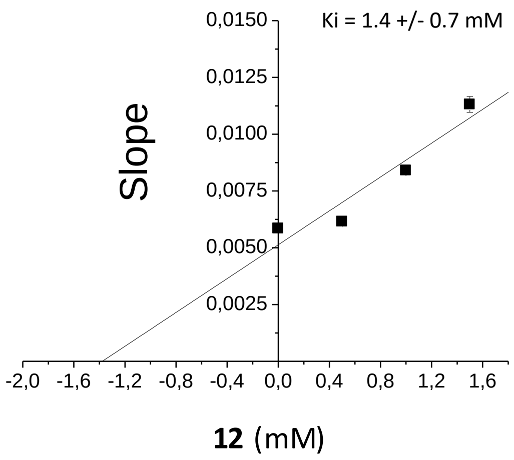

Figure S48: Plots for the determination of the Ki values of compound **12**.

Pharmacological Chaperoning Activity.

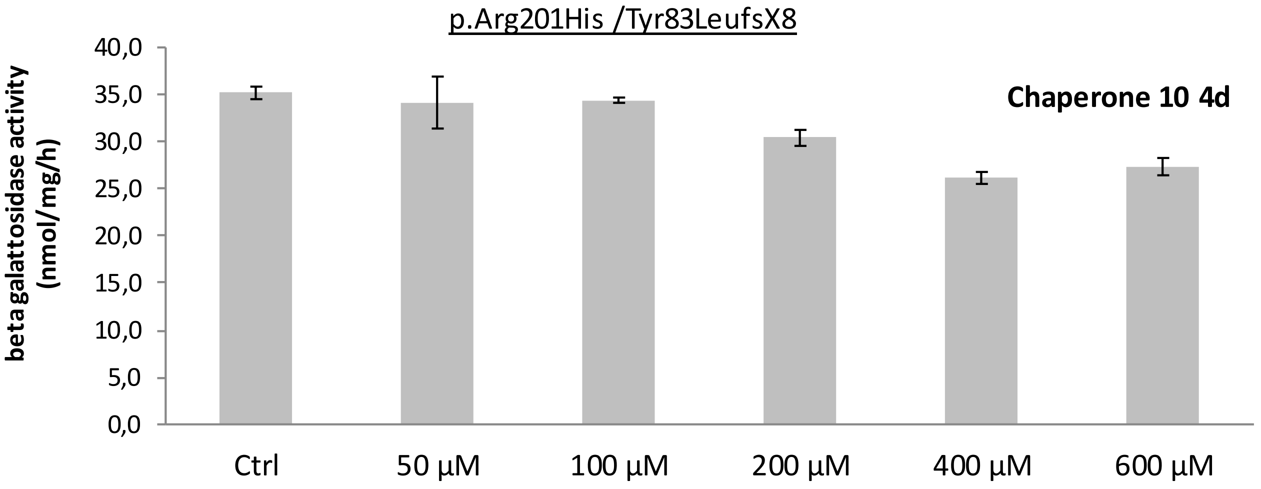

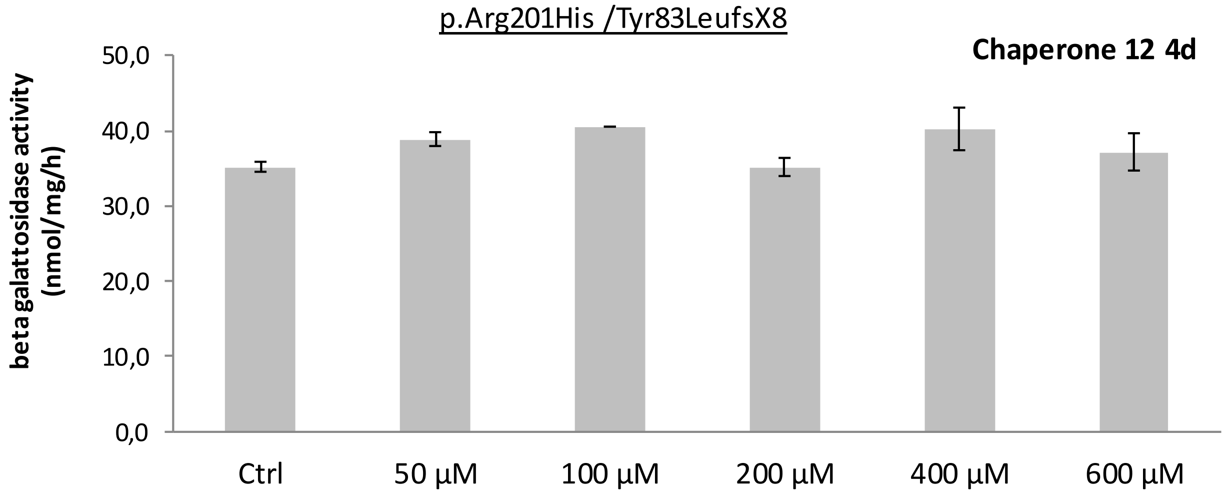

Figure S49: Fibroblasts derived from GM1 patients bearing the p.Arg201His /Tyr83LeufsX8 mutations were incubated without (control, ctrl) or with 5 different concentrations (50 µM, 100 µM, 200 µM, 400 µM, 600 µM) of compounds **10** and **12**. After 4 days, the β-gal activity was determined (as above described) in lysates from treated fibroblasts. Reported data are mean S.D. (n=2).

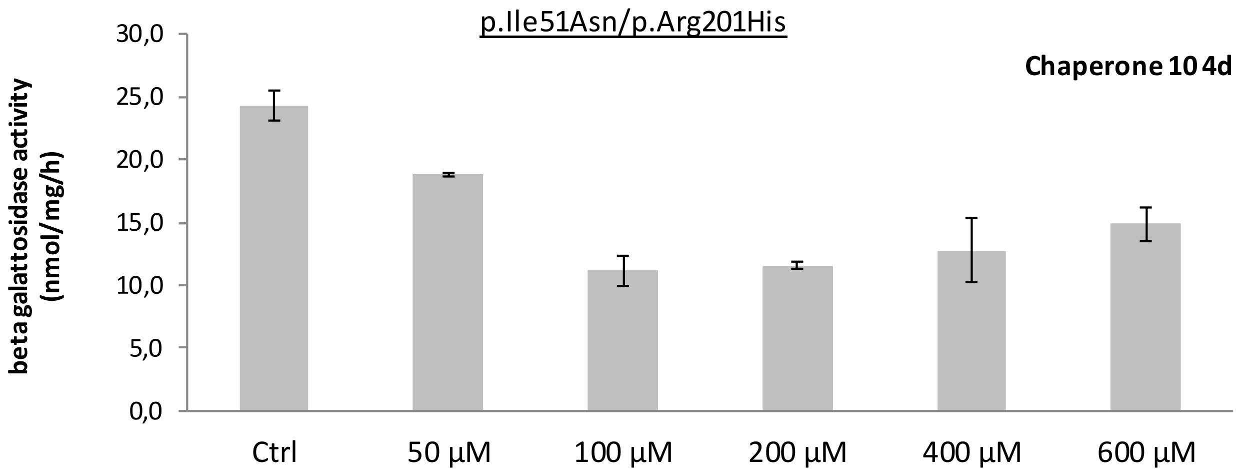

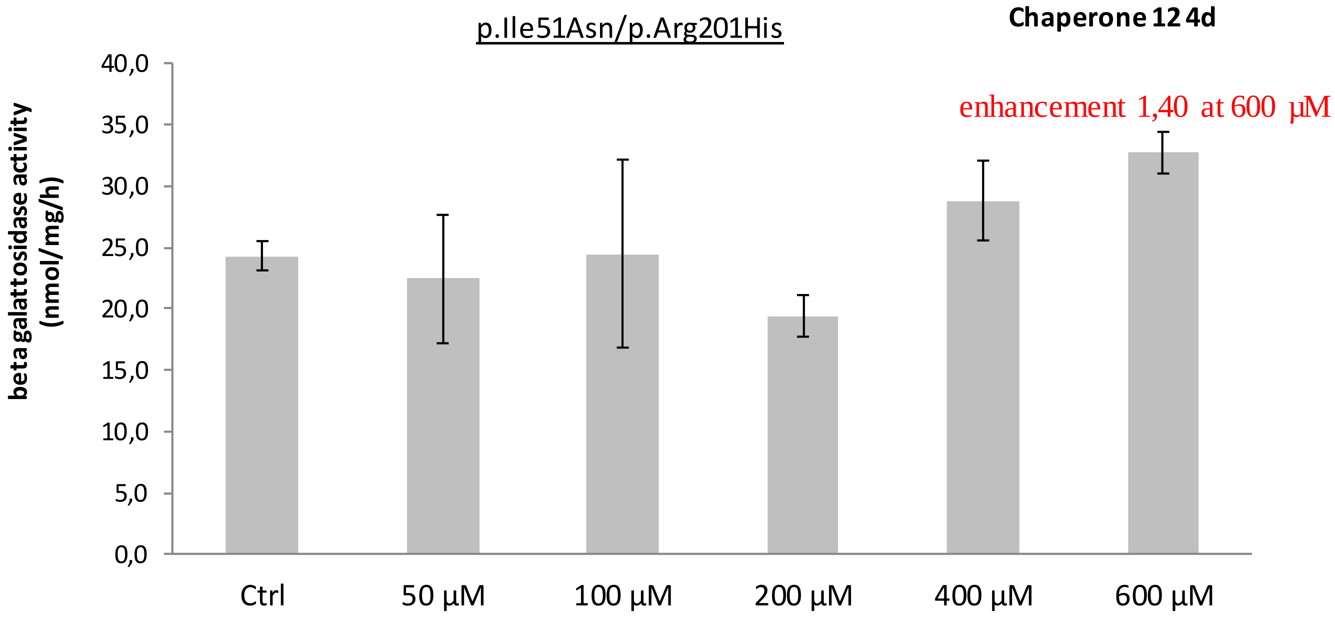

Figure S50: Fibroblasts derived from GM1 patients bearing the p.Ile51Asn/p.Arg201His mutations were incubated without (control, ctrl) or with 5 different concentrations (50 µM, 100 µM, 200 µM, 400 µM, 600 µM) of compounds **10** and **12**. After 4 days, the β-gal activity was determined (as above described) in lysates from treated fibroblasts. Reported data are mean S.D. (n=2).
